# Neutrophil–lymphocyte ratio predicts short term mortality in patients with hepatitis B virus-related acute-on-chronic liver failure treated with an artificial liver support system

**DOI:** 10.1371/journal.pone.0175332

**Published:** 2017-04-20

**Authors:** Zeng Fan, Chen EnQiang, Du Ling Yao, Yan LiBo, Li Hong, Bai Lang, Feng Ping, Tang Hong

**Affiliations:** 1 Centre of Infectious Diseases, West China Hospital, Sichuan University; 2 Department of intensive care unit, Hospital of the University of Electronic Science, Sichuan Provincial People’s Hospital, Chengdu, Sichuan, China; Yonsei University College of Medicine, REPUBLIC OF KOREA

## Abstract

**Aim:**

Hepatitis B virus-related acute-on-chronic liver failure has high short-term mortality. Artificial liver support systems (ALSS) may improve outcome and avoid liver transplantation, but predicting short-term prognosis in such patients is difficult. This study aimed to determine whether the neutrophil–lymphocyte ratio (NLR), an inflammation marker, predicted mortality in patients treated with ALSS.

**Methods:**

A total of 560 patients with hepatitis B virus-related acute-on-chronic liver failure were enrolled, 338 were treated with ALSS and the others treated with standard of care(SOC). Clinical variables and the NLR were evaluated for prognostic value.

**Results:**

Thirty-day mortality was 28.4% in ALSS and 55.4% in SOC patients. The NLR was lower in survivors than in ALSS or SOC patients who died. Univariate and multivariate analysis found that NLR and the chronic liver failure sequential organ failure assessment scores(CLIF-SOFA) were independently associated with 30-day mortality. Among patients with NLRs ≤ 3, 3–6, and >6, 30-day mortality was 7.7%,23.1%, and 69.2% in ALSS and 25.5%, 50.0%, and 75.0% in SOC patients. Among patients with NLRs ≤ 3 or 3–6, mortality was lower in ALSS than in SOC patients (*P* < 0.01). Mortality rates of ALSS and SOC patients with NLRs > 6 did not different (*P* >0.05). The area under curve of NLR and CLIF-SOFA was 0.82 and 0.88 in ALSS group, 0.78 and 0.86 in SOC group. The results suggest that liver function in most patients with NLRs ≤ 3 recovered with ALSS treatment, and patients with NLRs > 6 needed emergency liver transplantation.

**Conclusion:**

NLR was an independent predictor of mortality in ALSS patients and may assist physicians in determining treatment options.

## Introduction

Acute-on-chronic liver failure is a devastating life-threatening condition associated with a high mortality. In China, most cases of ACLF are caused by hepatitis B virus (HBV) infection.[1] The mortality of ACLF ranged 30% to 70% before liver transplantation (LT) was available.[2] Loss of hepatic synthetic and metabolic functions results in accumulation of molecular toxins such as ammonia and bile acids, mediators of inflammation, and endotoxins in the blood of ACLF patients. The consequent inflammatory reactions aggravate liver injury and can lead to encephalopathy and multiple organ dysfunction. Previous studies found that artificial liver support systems (ALSS) could remove toxic substances, correct severe coagulopathy, and assist in restoration of the regenerative functions of liver cells.[3] Although ALSS may improve the prognosis of ACLF patients and allow for resolution without LT, it is not always the effective treatment for every patient. For some patients, liver transplantation is the only choice. The management of ACLF is difficult because of lack of prognostic criteria and an incomplete understanding of the role and the timing of LT. A clinical experience report indicated that about 37% of patients who were listed for emergency LT recovered with ALSS and 50% died while waiting for an organ.[4] This has serious implications for both allocation of scarce donor organs and risks associated with unnecessary transplantation. It is essential to discriminate patients who require LT and those would survive with ALSS treatment only, so that the most urgent cases are identified. The Neutrophil–lymphocyte ratio (NLR), a marker of systemic inflammation, is a valuable prognostic marker in ACLF patients not treated with ALSS.[5] Previous studies have reported that liver cell necrosis led to release of inflammatory cytokines, triggering of immune responses, abundant movement of granulocytes into the peripheral blood from the bone marrow, and a decrease in lymphocyte number.[6, 7] To our knowledge, the utility of the NLR as a predictor of death in ACLF patients treated with ALSS has not been investigated. The goal of this study was to determine whether the NLR could predict the prognosis of patients with HBV–ACLF treated with ALSS.

## Methods

### Patient selection

We recruited 621 patients with hepatitis B virus-related acute-on-chronic liver failure (HBV–ACLF) who were admitted to West China hospital between February 2013 and January 2015. 61 patients were excluded(4 patients were hepatitis A,2 patients were hepatitis E,15patients were liver cancer,8 patients were autoimmune hepatitis,6 patients were drug-induced hepatitis,26 patients were alcohol-related liver disease. At last, 560 patients were included. Of these patients, 338 were treated with ALSS combined with standard of care(ALSS group) and 222 were given only standard of care (SOC group). The Ethics Committee of West China Hospital approved the study. Our study complies with the Declaration of Helsinki. To participate in this study, patients had to give written informed consent. Patients confected with human immunodeficiency virus, hepatitis A, C, D, or E virus, or with alcohol-related liver disease, autoimmune hepatitis, drug-induced hepatitis, or liver cancer were excluded. Baseline data including age, gender, laboratory and virology test results, and presence of major complications, were collected at the time of admission.

### Definitions of clinical variables

ACLF was diagnosed following the consensus recommendations of the Asian Pacific Association for the Study of the Liver (APASL).[8] Hepatic encephalopathy (HE) was defined by the HE scoring algorithm.[9] Hepatorenal syndrome (HRS) and spontaneous bacterial peritonitis (SBP) were defined by International Ascites Club criteria.[10] The HBV–DNA load was quantified by real-time polymerase chain reaction and had a lower limit of detection of 1000 IU/mL. The Model End-stage Liver Disease (MELD) score was calculated from the creatine and bilirubin concentrations and the international normalized ratio (INR):

MELDscore=9.57(lncreatinine)+3.78(lnbilirubin)+11.20(lnINR)+6.43,

where creatinine and bilirubin are measured in mg/dL.[11] The chronic liver failure sequential organ failure assessment (CLIF-SOFA) scores was based on six organ systems[12].

### Treatment schedule

Standard medical treatment (SOC) included energy supplements, prophylaxis against bacterial infections, treating complications, and antiviral therapy. The ALSS consisted of combined plasma exchange (PE) and bilirubin adsorption (BA) and was performed using a Diapact Braun continuous renal replacement therapy (CRRT) machine and bilirubin absorbent column (HB-H-6, ZiBo China). Vascular access was achieved by insertion of a double lumen dialysis catheter into the femoral vein or internal jugular vein. Heparin was used for anticoagulation, the blood flow rate during dialysis was 180±20 mL/min, and the total volume of exchanged fresh plasma was approximately 1500 ml. Patients were given two or three ALSS sessions per week. The ALSS sessions were stopped depending on improvement of clinical symptoms(patients without sick, emesis, HE) and liver function (the bilirubinemia with serum total bilirubin(TBIL)< 140umol/L, coagulopathy with INR <1.6or plasma prothrombin activity>40% within 48 hours). The primary endpoint was mortality at 30 days. Survival data was obtained from electronic medical records. Patients were followed-up by telephone for 30 days.

### Statistical analysis

Normally distributed and nonparametric continuous variables were compared using Student’s *t*-test and the Mann–Whitney test, respectively. The chi-squared test was used for comparisons of categorical data values. The t-test, Mann-Whitney test and chi-squaretest were used to conduct univariate analysis. Multivariate analysis was performed by logistic regression. Receiver-operator curves (ROC) were used to assess the accuracy of variables in predicting death at 30 days. We used the Kaplan–Meier method to perform survival analysis for different cut-off values. All data were analyzed using SPSS software (version 22.0; SPSS, Chicago, IL, USA). The results were expressed as means±standard deviation (SD) or medians and inter-quartile range (IQR). *P*-values less than 0.05 were considered to be statistically significant.

## Results

The characteristics of the patients before treatment are shown in Table 1. The Male gender, alanine aminotransferase (ALT), bilirubin, ammonia, hemoglobin, alpha fetoprotein (AFP) and the rate of antiviral therapy were higher, and age, creatinine and NLR were lower in the ALSS group than in the SOC group. The two groups had similar median MELD scores and CLIF-SOFA scores.The dataset for this study is also available as S1 File.

**Table 1:** Baseline characteristics of patients.

Variable	ALSS group (N = 338)	SOC group (N = 222)	*P* value
Age (years)	42.1 (11.5)	49.2 (13.2)	0.00
Male gender	312 (92.3)	185 (83.3)	0.00
ALT (IU/L)	246.0 (108.5–713.5)	159.0 (57.0–431.2)	0.01
AST (IU/L)	231.0 (119.0–545.0)	195.5 (91.7–404.7)	0.41
Bilirubin (mg/dL)	22.4 (15.9–28.1)	19.6 (13.1–27.0)	0.00
Blood ammonia (umol/L)	72.0 (50.0–102.5)	62.0 (47.0–92.0)	0.03
Creatinine (mg/dL)	0.89 (0.77–1.01)	0.97 (0.81–1.3)	0.00
INR	2.0 (1.7–2.5)	2.0 (1.6–2.5)	0.62
Hemoglobin (g/L)	130.0 (116.0–143.0)	122.0 (96.0–135.0)	0.00
Platelets (*10^9^/L)	97.5 (68.0–128.0)	88 (58.2–117.7)	0.05
NLR	3.7 (2.7–6.2)	4.9 (3.1–8.7)	0.00
MELD score	24.9 (21.9–28.9)	25.0 (20.0–30.0)	0.88
CLIF-SOFAscore	9.2±2.1	9.0±3.0	0.76
AFP (ng/ml)	45.7 (15.0–148.5)	16.4 (8.5–58.5)	0.00
HBV–DNA (log)	3.5 (3.0–5.3)	3.59 (3.0–5.7)	0.24
Cirrhosis	222 (65.7)	141 (63.5)	0.60
HE (grade≥2)	37 (10.9)	34 (15.3)	0.13
HRS	19 (5.6)	29 (13.1)	0.00
Inflammation			
Pneumonia	8 (2.3)	6 (2.6)	
SBP	98 (29.0)	70 (31.5)	0.52
urinary tract infection	3 (0.9)	2 (0.9)	
Antiviral therapy	335(99.1)	191(86.0)	0.00
ADV	74(21.9)	30(13.5)	
ETV	137(40.5)	81(36.5)	
LAM	91(26.9)	57(25.7)	
LdT	5(1.5)	3(1.4)	
ADV+ETV/LAM+ADV	23(6.8)	17(7.7)	
Interferon	5(1.5)	3(1.4)	

Values are expressed as mean (standard deviation), number (percent), or median (interquartile range)

ALT, alanine aminotransferase; AST, aspartate aminotransferase; INR, international coagulation; MELD

Model for End-Stage Liver Disease; AFP, alpha fetoprotein; HE, hepatic encephalopathy; SBP:

spontaneous bacterial peritonitis; HRS, hepatorenal syndrome; CI, confidence interval; ADV, adefovir; ETV, entecavir; LAM, lamivudine; LdT, telbivudine.

### Neutrophil–lymphocyte ratio was positively correlated with mortality of hepatitis B virus-related acute-on-chronic liver failure patients

**Fig 1:**
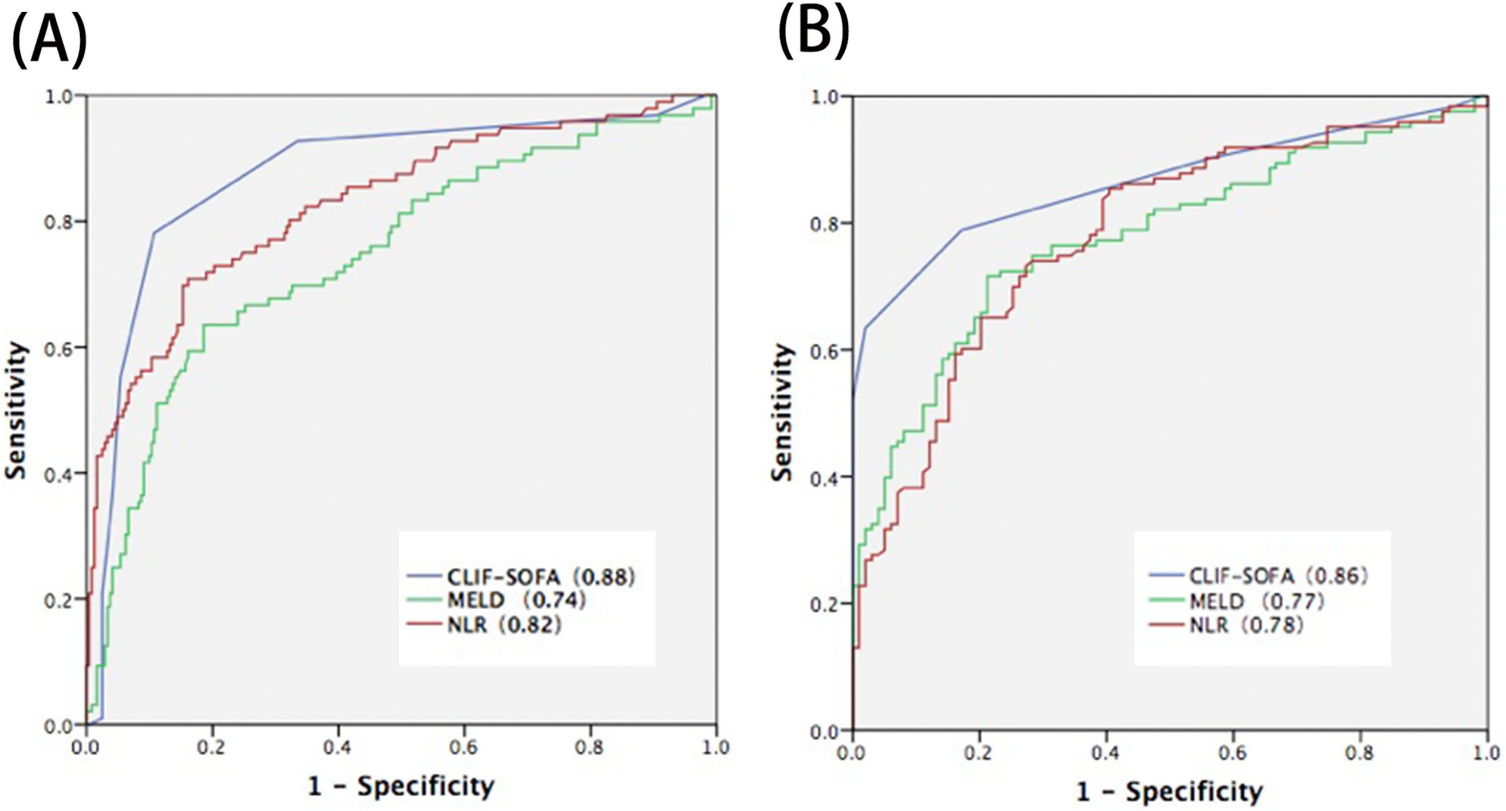
ROC of prognostic variables for patients with HBV-ACLF in ALSS (A) and SOC(B) group.

Variables associated with 30-day mortality on univariate and multivariate analysis of the whole cohort including ALSS group and SOC group was presented in Table 2. We found NLR was independently associated with 30 days mortality (HR 1.35;95%CI 1.24–1.46, p = 0.00).Other factors were ALSS therapy(HR 5.38,CI95%2.88–10.05), CLIF-SOFA score (HR 2.04,CI 95% 1.6–2.53), HBV-DNA (HR 0.82, 95CI% 0.69–0.96,p = 0.01).In the ALSS group, the 30-day mortality rate was lower than in the SOC group (28.4% vs 55.4%, *P* < 0.05). The results of univariate and multivariate analysis of the association of clinical variables with 30-day mortality in ALSS group or SOC group was shown in Tables 3 and 4. Univariate analysis found that higher NLR, total bilirubin, ammonia, creatinine, INR,MELD score, CLIF-SOFAscore, HE and HRS were predictors of mortality at 30 days in both study groups. Multivariate analysis found that NLR and CLIF-SOFA score were associated with worse mortality in both groups. To compare the predictive values of MELD score, CLIF-SOFA score and NLR in estimating the prognosis of patients with HBV-ACLF, we examined the ROC curve of these parameters (Fig 1). The area under curve(AUC) of NLR and CLIF-SOFA was 0.82 and 0.88 in ALSS group, 0.78 and 0.86 in SOC group.

**Table 2:** Univariate and multivariate analyses of variables affecting 30-day mortality in whole patients.

	Survival group (N = 341)	Death group (N = 219)	Univariate odds ratio (95% CI)	*P* value	Multivariate odds ratio (95% CI)	*P* value
Age (years)	43.3 (11.9)	47.4 (12.9)	-6.2 to -1.9	0.0		0.57
Male gender	309 (90.6)	188 (85.8)	-0.1 to 0.0	0.08		
ALSS	242 (71)	96 (43.8)	-0.3 to -0.2	0.00	5.38(2.88–10.05)	0.00
ALT (IU/L)	221.0(96.5–639.0)	174.0 (79.0–559.0)	-74.5 to 125.8	0.62		
AST (IU/L)	214.0 (102.0–482.0)	222.0 (112.0–492.0)	-159.2 to 42.0	0.25		
Bilirubin (mg/dL)	20.0 (13.9–25.4)	23.9 (16.2–30.5)	-5.1 to-1.9	0.00		0.68
Blood ammonia (umol/L)	63.0 (45.0–92.0)	76.0(51.0–112.0)	-22.7to-8.0	0.00		0.24
Creatinine (mg/dL)	0.88 (0.7–1.1)	1.0 (0.81–1.47)	-0.6 to-0.3	0.00		0.91
INR	1.9(1.6–2.4)	2.37 (1.9–3.1)	-1.9 to-0.4	0.00		0.35
Hemoglobin (g/L)	128.0 (112.0–142.5)	125.0 (102.0–139.0)	2.8to10.9	0.00		0.33
Platelets (*10^9^/L)	99.0 (69.5–128.5)	88.0 (58.0–124.0)	-4.6 to13.8	0.33		
NLR	3.4 (2.3–4.4)	7.2(4.2–11.7)	-5.5 to-4.1	0.00	1.35(1.24–1.46)	0.00
MELD score	23.4(20.5–26.5)	28.8 (24.3–35.0)	-7.2 to -4.8	0.00		0.47
CLIF-SOFAscore	8.2±1.6	11.1±2.7	-3.2 to -2.5	0.00	2.04 (1.64–2.53)	0.00
AFP (ng/ml)	46.3 (14.0–150.3)	17.5(11.0–56.8)	34.3to87.5	0.00	NS	0.33
HBeAg positive	129 (37.8)	73 (33.3)	-0.03o-0.1	0.28		
HBV–DNA (log)	3.9 (3.0–5.8)	3.0 (3.0–4.9)	0.18 to 0.72	0.00	0.82(0.69–0.96)	0.01
Cirrhosis	210 (61.6)	153 (69.9)	-0.16 to -0.00	0.05		
HE (grade ≥2)	16(4.7)	55 (25.1)	-0.26 to -0.15	0.00		0.81
SBP	86(25.2)	82 (37.4)	-0.20to -0.04	0.00		0.93
HRS	9 (2.6)	39 (17.8)	-0.19 to -0.1	0.00		0.34
Antiviral therapy	329 (96.5)	200 (91.3)	-0.09 to -0.01	0.01		0.51
ADV	68 (19.9)	36(16.4)				
ETV	141 (41.3)	78(35.6)				
LAM	90 (26.4)	58(26.5)				
LdT	6 (1.8)	2(0.9)				
ADV+ETV/LAM+ADV	21 (6.2)	21(9.6)				
Interferon	3 (0.9)	5(2.3)				

Values are expressed as mean (standard deviation), number (percent), or median (interquartile range)

ALT, alanine aminotransferase; AST, aspartate aminotransferase; INR, international coagulation; MELD

Model for End-Stage Liver Disease; CLID-SOFA, chronic liver failure sequential organ failure assessment score, AFP, alpha fetoprotein; HE, hepatic encephalopathy; SBP:

spontaneous bacterial peritonitis; HRS, hepatorenal syndrome; CI, confidence interval;ADV, adefovir; ETV, entecavir; LAM, lamivudine; LdT, telbivudine.

**Table 3:** Univariate and multivariate analyses of variables affecting 30-day mortality in the ALSS group.

	Survival group (N = 242)	Death group (N = 96)	Univariate analysis		Multivariate analysis	
			(95% CI)	*P* value	odds ratio (95% CI)	*P* value
Age (years)	41.9 (11.0)	42.2 (11.45)	-2.89 to 2.40	0.85		
Male gender	223 (92.1)	89 (92.7)	-0.58 to 0.69	0.86		
ALT (IU/L)	239.5 (109.0–724.2)	248.0 (96.0–677.7)	-142.5to141.6	0.93		
AST (IU/L)	222.0 (113.0–547.0)	246.0 (128.7–534.0)	-129.3 to 114.7	0.90		
Bilirubin (mg/dL)	20.7 (15.1–26.6)	26.0 (19.8–31.5)	-6.57to-2.39	0.00	NS	0.15
Blood ammonia (umol/L)	67.0 (46.5–98.5)	86.5 (59.2–124.0)	-30.53 to -10.27	0.00	NS	0.16
Creatinine (mg/dL)	0.88 (0.7–1.0)	0.93 (0.80–1.16)	-0.33to-0.12	0.00	NS	0.52
INR	2.0 (1.65–2.37)	2.6 (2.0–3.3)	-2.20to-0.39	0.00	NS	0.71
Hemoglobin (g/L)	129.0 (116.0–143.2)	134.0 (116.5–143.0)	-4.56to5.43	0.43		
Platelets (*10^9^/L)	100.0 (72.5–129.2)	90.0 (59.5–122.2)	-10.45to 14.47	0.75		
NLR	3.4 (2.4–4.3)	7.7 (4.2–11.8)	-5.71to-4.15	0.00	1.45 (1.28–1.65)	0.00
MELD score	24.0 (21.3–26.9)	29.9 (24.5–33.3)	-6.89to-3.99	0.00	NS	0.91
CLIF-SOFAscore	8.5±1.6	11.1±2.2	-3.01to-2.15	0.00	1.73(1.36–2.21)	0.00
AFP (ng/ml)	51.6 (15.1–152.2)	34.6 (14.6–131.0)	-6.2to79.2	0.09		
HBeAg positive	90 (37.2)	35 (36.5)	-0.11to 0.12	0.90		
HBV–DNA (log)	4.1 (3.0–5.9)	3.0 (3.0–3.1)	0,75to1.44	0.00	0.54(0.40–0.71)	0.00
Cirrhosis	151 (62.3)	69 (71.9)	-0.19 to 0.03	0.13		
HE (grade ≥2)	14 (5.8)	23 (24.0)	-0.25 to-0.11	0.00	NS	0.63
SBP	61 (25.2)	37 (38.5)	-0.24 to -0.03	0.01	NS	0.74
HRS	7 (2.9)	12 (12.5)	-0.15 to -0.04	0.00	NS	0.63
Antiviral therapy	242 (100)	96 (100)		1		
ADV	52 (21.5)	22 (22.9)				
ETV	106 (43.8)	32 (33.3)				
LAM	63 (26.0)	28 (29.2)				
LdT	4 (1.7)	1 (1.0)				
ADV+ETV/LAM+ADV	14 (5.8)	11 (11.5)				
Interferon	3 (1.2)	2 (2.1)				

Values are expressed as mean (standard deviation), number (percent), or median (interquartile range)

ALT, alanine aminotransferase; AST, aspartate aminotransferase; INR, international coagulation; MELD

Model for End-Stage Liver Disease; CLID-SOFA,chronic liver failure sequential organ failure assessment score, DAFP, alpha fetoprotein; HE, hepatic encephalopathy; SBP:

spontaneous bacterial peritonitis; HRS, hepatorenal syndrome; CI, confidence interval; ADV, adefovir; ETV, entecavir; LAM, lamivudine; LdT, telbivudine.

**Table 4:** Univariate and multivariate analyses of variables affecting 30-day mortality in the SOC group.

	Survival group (N = 99)	Death group (N = 123)	Univariate analysis		Multivariate analysis	
			(95% CI)	*P* value	odds ratio (95% CI)	*P* value
Age (years)	46.5 (13.3)	51.4 (12.7)	-8.38to-1.46	0.00	NS	0.05
Male gender	86 (86.9)	99 (80.5)	-0.16 to -0.03	0.20		
ALT (IU/L)	166.0 (60.0–446.0)	155.0 (55.0–430.0)	-180.1 to139.3	0.80		
AST (IU/L)	182.0 (87.0–371.0)	198.0 (97.0–474.0)	-343.5 to 23.3	0.08		
Bilirubin (mg/dL)	17.3 (10.5–23.0)	22.0 (14.6–29.5)	-7.26 to -2.1	0.00	NS	0.22
Blood ammonia (umol/L)	57.0 (41.0–77.5)	69.0 (50.0–104.5)	1–28.45 to-5.48	0.00	NS	0.97
Creatinine(mg/dL)	0.89 (0.79–1.05)	1.11 (0.84–1.53)	-0.93to -0.34	0.00	NS	0.07
INR	1.8 (1.6–2.0)	2.3 (1.8–3.0)	-2.8 to-0.04	0.00	8.87(1.16–68.08)	0.04
Hemoglobin (g/L)	126.0 (105.0–140.0)	117.0 (91.0–130.0)	1.16 to15.42	0.02	NS	0.98
Platelets (*10^9^/L)	95.0 (58.0–123.0)	84.0 (52.0–128.0)	-12.79to17.48	0.76		
NLR	3.70 (2.37–5.50)	6.6 (4.1–11.3)	-5.83 to-3.23	0.00	1.28 (1.13–1.46)	0.00
MELD score	21.60 (17.90–24.8)	28.5 (24.0–35.3)	-10.08to-5.91	0.00	0.66(0.48–0.91)	0.00
CLIF-SOFAscore	7.5±1.2	11.1±3.1	-4.27 to-2.96	0.00	3.37 (1.93–5.89)	0.00
AFP (ng/ml)	30.9 (18.11–156.95)	15.28 (3.9–42.6)	37.45 to 93.92	0.00	NS	0.62
HBeAg positive	39 (39.4)	38 (30.9)	-0.04 to0.21	0.18		
HBV–DNA (log)	3.74 (3.00–5.59)	3.3 (3.0–6.0)	-0.60to2.87	0.48		
Cirrhosis	57 (57.6)	84 (68.3)	-0.23to0.02	0.10		
HE (grade≥2)	2 (2.0)	32 (26)	-0.33 to-0.15	0.00	NS	0.39
SBP	25 (25.3)	45 (36.6)	-0.23 to0.01	0.07		
HRS	2 (2.0)	27 (22)	-0.28 to-0.11	0.00	NS	0.46
Antiviral therapy	87 (87.9)	104(84.6)	-0.13 to0.05	0.35		
ADV	16 (16.2)	14(11.4)				
ETV	35 (35.4)	46(37.4)				
LAM	27 (27.3)	30(24.4)				
LdT	2 (2.0)	1(0.8)				
ADV+ETV/LAM+ADV	7 (7.1)	10 (8.1)				
Interferon	0 (0)	3(2.4)				

Values are expressed as mean (standard deviation), number (percent), or median (interquartile range)

ALT, alanine aminotransferase; AST, aspartate aminotransferase; INR, international coagulation; MELD

Model for End-Stage Liver Disease; CLID-SOFA, chronic liver failure sequential organ failure assessment score, AFP, alpha fetoprotein; HE, hepatic encephalopathy; SBP:

spontaneous bacterial peritonitis; HRS, hepatorenal syndrome; CI, confidence interval;ADV, adefovir; ETV, entecavir; LAM, lamivudine; LdT, telbivudine.

### Neutrophil–lymphocyte ratio as a prognostic marker for patients treated with an artificial liver support system

**Fig 2:**
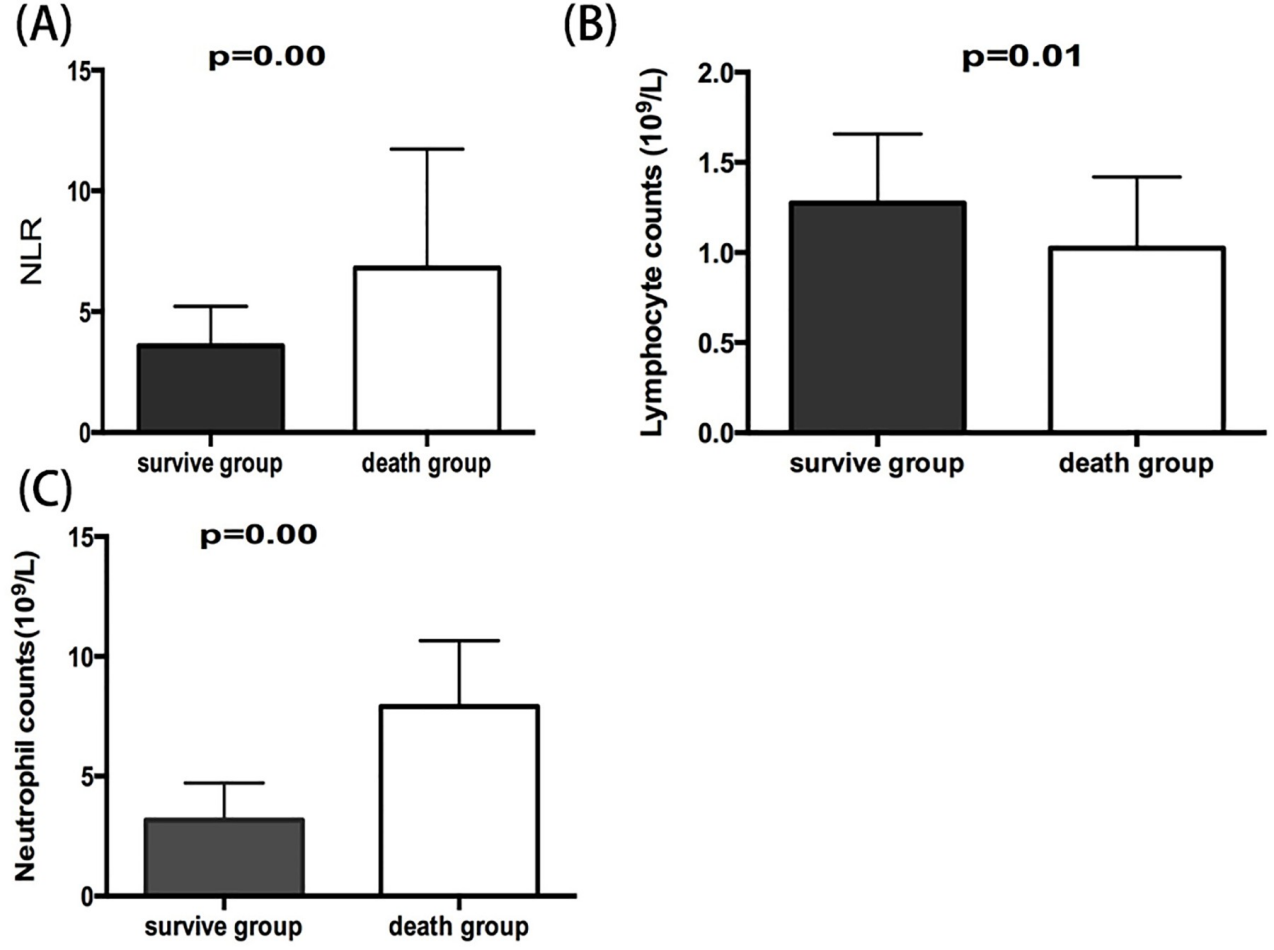
Baseline median NLR (A), LC (B), and NC (C) in the ALSS group survivors (solid bars) and in patients who died (open bars). Error bars are IQRs.

In the ALSS group, the median NLR and neutrophil count (NC) were higher in patients who died than in survivors [7.7 (4.2–11.8) vs. 3.4 (2.4–4.3), and 7.9 (4.4–10.6) vs. 3.2 (2.5–4.7), respectively, both *P* = 0.00], and the lymphocyte count (LC) was lower in patients who died [0.9 (0.7–1.5)] than in survivors [1.2 (0.9–1.6), *P* = 0.01] (Fig 2).

**Fig 3:**
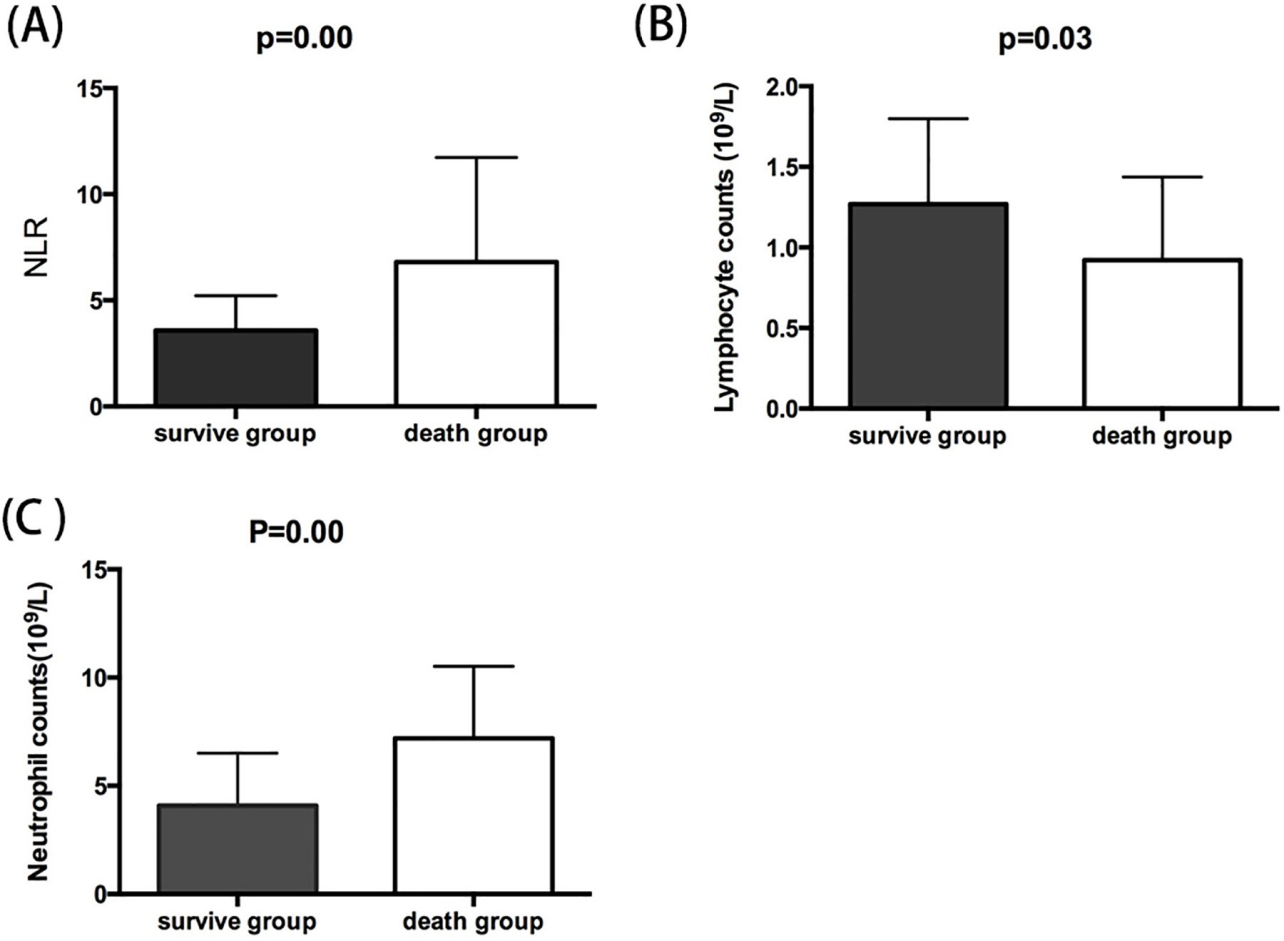
Baseline median NLR (A), LC (B), and NC (C) in the SOC group survivors (solid bars) and in patients who died (open bars). Error bars are IQRs.

In the SOC group, the median NLR and NC were higher in patients who died [6.8 (4.2–11.7) vs. 3.6 (2.3–5.2), and 7.2 (5.2–10.5) vs. 4.1 (2.8–6.5), both *P* = 0.00], and the LC was lower in patients who died [1.0 (0.7–1.5)] than in survivors [1.2 (IQR 0.8–1.8, *P* = 0.03) (Fig 3).

**Fig 4:**
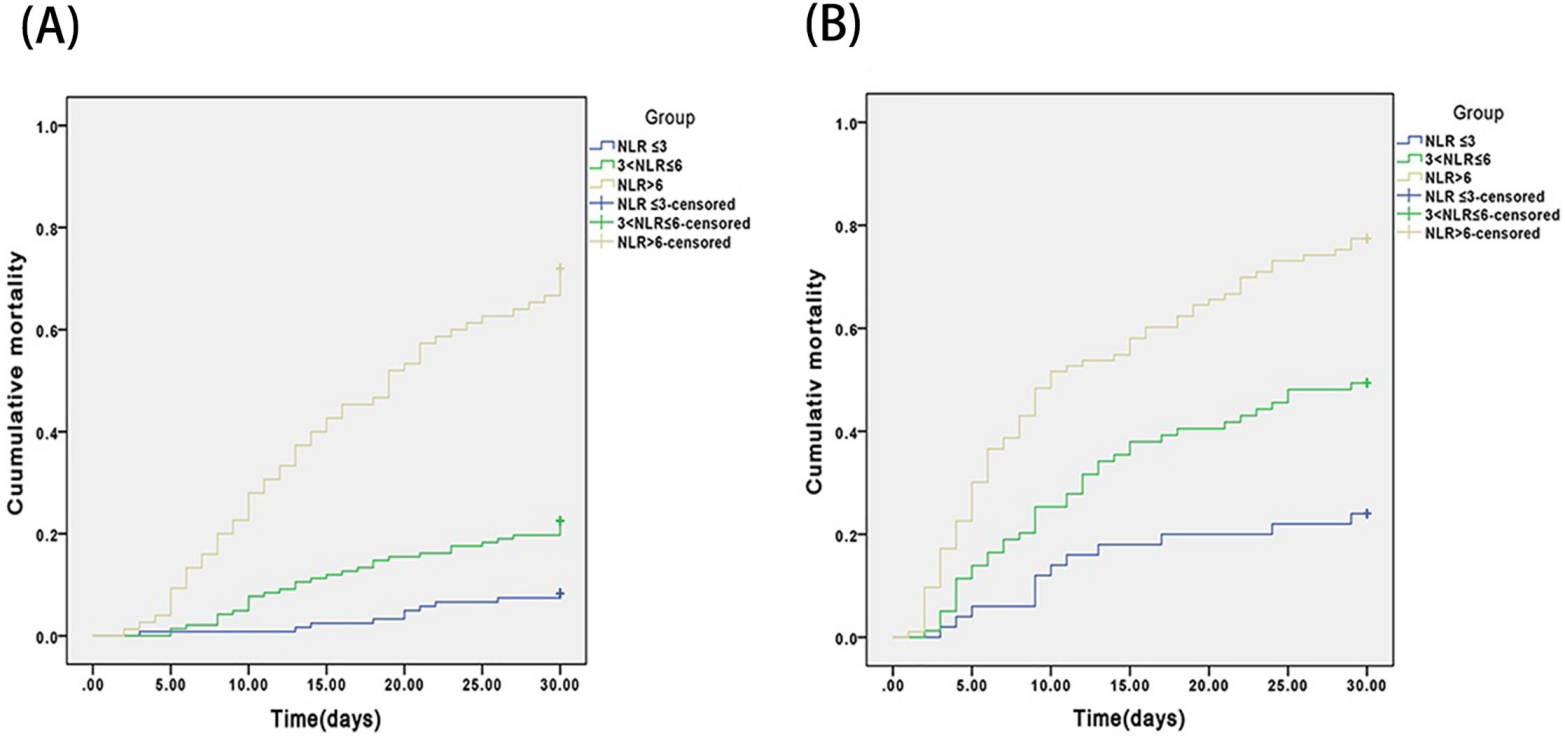
Cumulative 30-day mortality in ALSS (A) and SOC (B) patients with NLR ≤ 3, 3–6, and > 6.

In the survival analysis of this sample of HBV–ACLF patients, a baseline NLR ≤ 3 was chosen as a negative cut-off value and a baseline NLR > 6 was chosen as a positive cut-off value. In the ALSS group, 7.7% of patients (9/117) with an NLR ≤ 3, 23.1% with an NLR of 3–6 (33/143), and 69.2% with an NLR > 6 (54/78) died within 30 days (Fig 4A). In the SOC group, 25.5% of patients (14/55) with an NLR ≤ 3, 50% with an NLR of 3–6 (29/58), and 75.0% with an NLR > 6 (80/109) died within 30 days (Fig 4B). The differences in 30-day mortality observed in the ALSS and SOC groups were significant for patients with an NLR ≤ 3 and those with an NLR from 3–6 (both *P* < 0.01). The between-group difference in 30-day mortality of patients with an NLR > 6 was not significant (*P* = 0.53). The mortality rates of ALSS patients with NLRs ≤ 3 and 3–6 were both lower than that of SOC patients with similar NLRs (*P* < 0.05). The survival analysis thus indicated that the prognosis of patients with NLRs ≤ 3 or 3–6 can be improved by an ALSS. The analysis also showed that patients with an NLR > 6 have a poor prognosis and need emergency LT.

## Discussion

We found that the NLR in peripheral blood, which is a marker of systemic inflammation, predicted prognosis in ACLF patients treated with an ALSS. In univariate and in multivariate analysis NLR was an independent predictor of 30-day mortality of patients in both the ALSS and SOC groups. In addition, the NLR was significantly higher (*P* < 0.01) in ALSS patients who died (7.7) than in those who survived (3.4). The difference the NLR of SOC patients who died (6.8) and those who survived (3.6) was also significant (*P* < 0.01). An NLR ≤ 3 predicted lower mortality and an NLR > 6 was a warning of high mortality risk. Kaplan–Meier survival analysis showed that each increase of NLR was associated with greater 30-day mortality. Comparison of the ALSS and SOC groups revealed that the mortality of patients with an NLR ≤ 3 or 3–6 was lower in the ALSS than in the SOC group, but that mortality was not different when the NLR was > 6. In the ALSS group, the mortality of patients with an NLR ≤ 3 was lower than in patients with NLR from 3–6. The 30-day mortality results thus indicate that ALSS improved the prognosis of patients with NLR ≤ 3 or 3–6. Liver function in most patients with NLR ≤ 3 recovered with ALSS treatment, Patients with an NLR > 6 had a poor prognosis and required emergency LT. The ability to estimate prognosis would make it easier for physicians to discuss the expected benefits of available treatments with the patient and to schedule the best therapy.

NLR is associated with prognosis in other diseases. Previous studies found that a high baseline NLR was associated with poor treatment response in patients with hepatocellular carcinoma (HCC)[13] and increased risk of mortality in patients with acute pancreatitis or acute coronary syndrome[14, 15]. A few studies have examined the role of NLR in ACLF, but all excluded patients who received ALSS[16]. To our knowledge, this is the first study to shown that the NLR was an independent predictor of mortality in ALSS-treated patients.

ALSS removes excess concentrations of toxic substances, correct severe coagulopathy, and provide an internal environment suitable for liver cells to restore liver functions[17].The major objective of ALSS is to buy time by doing some of the work of the liver. However, if liver necrosis is too severe, then there may not be enough liver cells for regeneration to occur even with ALSS. Inflammation plays an important role in the development and progression of ACLF, as shown by the accumulation of activated blood neutrophils in the liver in response to release of multiple proinflammatory cytokines. The activated neutrophils degranulate and release oxidants that diffuse into hepatocytes and trigger intracellular oxidative stress and mitochondrial dysfunction[18]. Our study observed significantly higher baseline neutrophil levels in patients who died than in those who survived. Several studies have found that the elevation of neutrophils in ACLF might be correlated with aggravated liver injury[19, 20], and others have found that, lymphocyte responses are important in viral control and immune-mediated liver damage. Zhou *et al* showed that the numbers of CD4^+^ and CD8^+^ T lymphocytes were significantly higher in patients with ACLF than those with chronic hepatitis B[21]. but an excessive inflammation response may thus lead to lymphocyte destruction or suppression. Therefore, a high NC together with a low LC may reflect the severity of liver failure, and the NLR may increase with the severity of liver damage.

Over the years, many clinical and biochemical parameters have been proposed as prognostic factors in ACLF patients. But most were suggested by data obtained from patients who were not given ALSS treatment. Evidence provided by recent studies shows that with proper timing, the use of ALSS can support liver function and decrease mortality without a need for LT. Thus, it is extremely important to distinguish patients who require LT from those who would survive with ALSS treatment only. In this study, we found that NLR was an independent predictor of mortality. In patients with a baseline NLR > 6, mortality in the ALSS group was more than 70%, which was similar to mortality in the SOC group, indicating that LT was necessary in those patients. By contrast, the 30-day mortality in ALSS patients with an NLR ≤ 3 was less than 10%, suggesting that LT was no longer necessary. ALSS treatment was continued in those patients. The mortality results supported the use of NLR as a prognostic marker that can assist physicians to accurately identify the patients who need LT.

This retrospective study was limited by data collected at a single center, which increases the likelihood of selection bias. Although our study included 560 patients who were treated over the past 4 years, additional, prospective studies with larger sample sizes are needed to confirm our results.

In conclusion, our study showed, for the first time, that the NLR was an independent predictor of mortality in ACLF patients treated with ALSS. The survival analysis identified cut-off values of the NLR that may help to predict patient mortality and to assist physicians in selecting the best therapy for individual patients.

## Supporting information

S1 File Dataset underlying the study. (PDF)
