# Supplementary material for: Neutrophil–lymphocyte ratio predicts short term mortality in patients with hepatitis B virus-related acute-on-chronic liver failure treated with an artificial liver support system
Source: PLoS One. 2017 Apr 20;12(4):e0175332. doi: 10.1371/journal.pone.0175332 (PMC5398520; doi:10.1371/journal.pone.0175332)
Supplement: S1 File — (PDF) [file pone.0175332.s001.pdf]

SOLF	30 daymo	SEX（1mal	year	alss（1ye	SBP（0 NO	HE（0 NO1	Cirrhosis	HRS（0 NO1
14.0	2.0	1	40.0	2	0	1	0	1
12.0	2.0	1	50.0	2	0	1	1	0
10.0	2.0	1	46.0	2	0	0	0	0
7.0	2.0	1	49.0	1	1	0	1	0
14.0	2.0	1	67.0	2	0	0	1	1
10.0	2.0	2	57.0	2	1	0	1	0
13.0	2.0	1	34.0	1	0	0	1	0
9.0	2.0	1	37.0	1	0	0	1	0
6.0	2.0	1	43.0	2	0	0	0	0
16.0	2.0	1	50.0	2	1	1	1	1
11.0	2.0	2	60.0	1	0	1	0	0
12.0	2.0	1	57.0	2	1	0	1	1
7.0	2.0	1	58.0	2	0	0	1	0
9.0	2.0	1	22.0	2	0	0	1	0
10.0	2.0	2	58.0	1	0	1	0	0
12.0	2.0	1	47.0	2	1	0	1	1
10.0	2.0	2	67.0	2	1	0	0	0
10.0	2.0	1	40.0	1	1	0	1	0
16.0	2.0	1	56.0	1	1	1	1	0
10.0	2.0	1	51.0	2	1	0	1	0
14.0	2.0	1	49.0	2	0	1	1	0
8.0	2.0	1	51.0	1	1	0	1	0
12.0	2.0	1	26.0	2	0	0	0	0
14.0	2.0	1	60.0	1	0	0	1	0
17.0	2.0	2	32.0	2	0	1	1	0
7.0	1.0	1	77.0	2	0	0	0	0
7.0	1.0	1	55.0	1	1	0	1	0
13.0	2.0	1	58.0	1	0	1	1	0
11.0	2.0	2	54.0	1	1	0	1	0
14.0	2.0	1	50.0	2	1	0	1	1
19.0	2.0	1	51.0	2	0	1	1	1
11.0	2.0	2	37.0	1	0	0	1	0
10.0	2.0	1	39.0	1	0	0	1	0
5.0	2.0	1	56.0	2	0	0	1	0
10.0	2.0	1	52.0	2	1	1	1	0
9.0	2.0	1	64.0	1	1	0	1	0
10.0	2.0	1	44.0	1	1	0	1	0
15.0	2.0	1	65.0	2	1	1	0	0
15.0	2.0	1	46.0	1	0	1	1	1
15.0	2.0	1	46.0	1	0	1	1	1
7.0	2.0	1	49.0	1	1	1	0	0
11.0	2.0	1	33.0	1	0	1	0	0
15.0	2.0	1	61.0	2	1	1	1	1
9.0	2.0	1	31.0	1	0	0	1	0
8.0	1.0	1	35.0	1	0	0	1	0

10.0	2.0	1	49.0	2	0	0	1	0
11.0	2.0	1	23.0	1	1	0	1	1
11.0	2.0	1	58.0	2	0	0	1	1
10.0	2.0	1	62.0	2	1	0	1	0
16.0	2.0	1	58.0	1	0	1	1	0
12.0	2.0	1	46.0	1	0	1	0	0
8.0	2.0	2	67.0	2	0	0	0	0
14.0	2.0	1	46.0	2	0	1	0	0
14.0	2.0	1	48.0	2	1	0	0	0
9.0	1.0	1	70.0	2	0	0	1	0
13.0	2.0	1	48.0	2	0	1	1	1
12.0	2.0	1	41.0	1	1	0	1	1
16.0	2.0	1	33.0	2	1	1	1	0
12.0	2.0	1	36.0	2	1	1	1	1
7.0	2.0	1	43.0	2	1	0	1	0
9.0	1.0	1	22.0	1	1	0	0	0
12.0	2.0	1	56.0	1	1	0	1	1
11.0	2.0	1	45.0	1	0	0	1	0
12.0	2.0	1	58.0	2	0	0	1	1
11.0	2.0	1	43.0	1	0	0	1	0
7.0	1.0	1	42.0	2	0	0	1	0
20.0	2.0	1	34.0	2	1	1	1	1
9.0	1.0	1	34.0	2	0	0	1	0
14.0	2.0	1	44.0	1	0	0	1	0
9.0	2.0	1	41.0	1	0	0	1	0
6.0	1.0	2	41.0	2	0	0	1	0
9.0	2.0	2	73.0	2	1	0	1	0
10.0	2.0	1	64.0	1	0	0	0	0
12.0	2.0	1	61.0	1	1	0	1	0
15.0	2.0	2	67.0	2	1	0	1	1
6.0	2.0	1	52.0	2	0	0	1	0
14.0	2.0	1	32.0	1	1	1	0	0
10.0	2.0	1	36.0	1	0	0	1	0
11.0	2.0	1	64.0	2	0	0	1	1
11.0	2.0	1	59.0	2	1	0	1	0
8.0	1.0	1	44.0	1	0	0	1	0
8.0	1.0	1	83.0	2	0	0	0	0
11.0	2.0	1	40.0	1	0	0	1	0
5.0	2.0	1	80.0	2	0	0	0	0
10.0	2.0	1	33.0	1	0	0	1	0
8.0	1.0	1	68.0	2	0	0	1	0
9.0	2.0	1	47.0	1	1	0	1	0
12.0	2.0	1	51.0	1	0	0	1	0
11.0	2.0	1	58.0	2	1	0	1	0
7.0	2.0	1	56.0	2	1	0	1	0
16.0	2.0	1	51.0	1	1	1	0	1

12.0	2.0	1	70.0	1	0	0	0	0
8.0	2.0	2	69.0	2	1	0	1	0
12.0	2.0	1	49.0	1	0	0	1	0
14.0	2.0	1	72.0	1	1	1	1	1
9.0	2.0	1	51.0	2	0	0	0	0
8.0	1.0	1	36.0	1	0	0	1	0
10.0	1.0	1	55.0	1	0	0	1	0
9.0	2.0	1	42.0	1	0	0	1	0
14.0	2.0	1	39.0	2	0	1	1	0
8.0	1.0	1	37.0	1	1	0	1	0
17.0	2.0	1	40.0	2	1	1	1	1
8.0	1.0	2	75.0	2	0	0	0	0
9.0	2.0	1	35.0	2	0	0	0	0
12.0	2.0	1	47.0	1	0	0	1	0
8.0	1.0	1	44.0	1	0	0	1	0
9.0	2.0	1	52.0	1	0	0	1	0
8.0	1.0	1	34.0	1	0	0	1	0
8.0	1.0	1	38.0	2	1	0	1	0
8.0	1.0	1	24.0	1	0	0	1	0
10.0	2.0	1	29.0	1	1	0	0	0
9.0	1.0	1	60.0	1	0	0	1	0
12.0	2.0	1	20.0	1	0	0	0	0
9.0	1.0	1	40.0	2	0	0	0	0
10.0	1.0	1	39.0	1	1	0	0	0
9.0	1.0	1	43.0	2	1	0	1	0
12.0	2.0	1	43.0	2	0	0	1	1
9.0	2.0	1	32.0	1	1	0	1	0
9.0	1.0	2	59.0	1	1	0	0	0
9.0	1.0	1	24.0	1	0	0	1	0
13.0	2.0	1	57.0	2	1	1	1	0
16.0	2.0	1	46.0	2	0	1	0	0
11.0	2.0	1	43.0	2	1	0	1	1
10.0	1.0	1	48.0	2	1	0	0	0
17.0	2.0	1	49.0	2	1	1	1	0
15.0	2.0	1	45.0	1	1	0	1	1
12.0	2.0	2	40.0	2	1	0	0	0
11.0	2.0	1	38.0	2	0	0	1	0
8.0	1.0	1	50.0	1	0	0	0	0
9.0	2.0	1	24.0	2	0	0	0	0
14.0	2.0	1	21.0	1	0	0	0	0
8.0	1.0	1	39.0	1	0	0	1	0
12.0	2.0	1	75.0	2	0	1	1	0
6.0	1.0	1	22.0	2	1	0	1	0
11.0	2.0	1	36.0	1	1	0	0	0
11.0	2.0	2	58.0	2	0	1	1	1
10.0	2.0	1	35.0	1	0	0	1	0

10.0	2.0	2	60.0	2	0	0	1	1
9.0	1.0	1	37.0	1	1	0	1	0
12.0	2.0	1	48.0	1	0	0	0	0
8.0	2.0	2	52.0	2	0	0	1	0
9.0	2.0	1	39.0	2	0	0	0	0
6.0	1.0	1	42.0	2	0	0	0	0
11.0	1.0	1	49.0	1	1	1	0	0
7.0	1.0	1	44.0	1	1	0	1	0
7.0	1.0	2	52.0	2	0	0	1	0
12.0	2.0	1	45.0	2	0	1	1	0
12.0	2.0	1	41.0	1	1	1	1	0
7.0	2.0	1	49.0	2	0	0	1	0
12.0	1.0	1	31.0	1	1	1	1	0
12.0	2.0	2	39.0	2	1	0	1	0
14.0	2.0	1	69.0	2	1	0	0	1
9.0	1.0	1	39.0	1	0	1	1	0
10.0	2.0	1	46.0	2	0	1	1	0
8.0	2.0	1	35.0	2	0	0	0	0
8.0	2.0	1	35.0	2	0	0	1	0
11.0	2.0	1	40.0	1	0	0	1	0
9.0	1.0	2	37.0	1	0	0	1	0
8.0	1.0	1	25.0	1	1	0	0	0
9.0	1.0	1	52.0	1	0	0	1	0
13.0	2.0	1	49.0	2	1	0	1	1
8.0	1.0	1	64.0	2	1	0	1	0
11.0	1.0	1	45.0	1	0	0	1	0
10.0	2.0	1	39.0	1	0	0	1	0
10.0	2.0	1	38.0	1	1	0	1	0
15.0	2.0	1	57.0	2	1	0	1	1
10.0	1.0	1	47.0	1	1	0	1	1
8.0	1.0	1	39.0	1	1	0	0	0
11.0	1.0	1	28.0	1	1	1	0	1
12.0	2.0	1	62.0	2	0	0	0	0
9.0	1.0	1	34.0	1	1	0	1	0
8.0	1.0	1	44.0	1	0	0	1	0
16.0	2.0	1	60.0	2	1	1	1	0
9.0	2.0	1	44.0	2	0	0	1	0
15.0	2.0	1	51.0	2	0	1	0	0
6.0	1.0	1	60.0	2	0	0	1	0
9.0	1.0	1	48.0	1	1	0	1	0
11.0	2.0	2	68.0	2	1	0	0	0
5.0	1.0	1	44.0	2	0	0	1	0
16.0	2.0	1	62.0	1	0	1	1	0
7.0	1.0	1	48.0	2	0	0	1	0
6.0	1.0	1	38.0	1	0	0	0	0
7.0	1.0	1	61.0	2	0	0	1	0

12.0	2.0	1	43.0	1	0	0	1	0
6.0	1.0	1	57.0	1	0	0	0	0
9.0	2.0	1	50.0	1	0	0	1	0
12.0	2.0	1	50.0	2	0	0	0	0
8.0	2.0	1	64.0	2	0	0	1	0
10.0	2.0	2	53.0	2	0	0	0	0
9.0	2.0	2	80.0	2	0	0	0	0
9.0	2.0	1	39.0	2	0	0	1	0
9.0	1.0	1	27.0	1	0	0	1	0
11.0	2.0	1	40.0	2	0	0	0	0
10.0	1.0	2	55.0	2	0	0	1	1
13.0	2.0	1	32.0	1	0	0	0	1
12.0	1.0	1	36.0	1	1	1	1	0
11.0	2.0	1	25.0	1	0	0	0	0
12.0	1.0	1	37.0	1	1	1	1	0
8.0	1.0	1	31.0	1	0	0	0	0
9.0	1.0	1	31.0	2	0	0	0	0
8.0	1.0	1	39.0	2	0	0	1	0
11.0	2.0	1	47.0	1	1	0	0	0
10.0	2.0	1	35.0	1	1	0	1	0
8.0	1.0	1	48.0	2	0	0	0	0
9.0	2.0	1	59.0	1	1	0	1	0
11.0	2.0	1	28.0	1	0	0	1	0
11.0	2.0	2	68.0	2	1	0	1	0
9.0	2.0	1	47.0	2	0	0	0	0
6.0	1.0	1	65.0	2	0	0	1	0
7.0	2.0	1	56.0	2	0	0	1	0
11.0	2.0	1	44.0	1	0	0	1	0
9.0	2.0	1	45.0	1	0	0	1	0
10.0	2.0	1	43.0	2	0	0	1	0
8.0	2.0	2	57.0	2	1	0	1	0
9.0	1.0	1	50.0	1	0	0	1	0
8.0	1.0	1	59.0	1	0	0	0	0
14.0	2.0	1	41.0	1	1	1	0	0
16.0	2.0	1	48.0	2	1	1	0	1
9.0	1.0	2	51.0	1	1	0	1	0
8.0	1.0	1	62.0	2	0	0	0	0
10.0	2.0	1	26.0	2	1	0	1	0
13.0	2.0	1	23.0	2	0	0	1	0
9.0	1.0	1	50.0	2	1	0	1	0
8.0	2.0	1	43.0	2	0	0	1	0
9.0	2.0	1	55.0	2	1	0	1	0
8.0	1.0	1	65.0	1	1	0	1	0
8.0	1.0	1	36.0	1	0	0	1	0
9.0	1.0	1	43.0	1	0	0	1	0
8.0	2.0	1	67.0	2	0	0	0	0

7.0	1.0	1	38.0	2	1	0	0	0
8.0	1.0	1	52.0	1	0	0	1	0
10.0	1.0	1	46.0	1	0	0	1	0
7.0	1.0	1	43.0	2	1	0	0	0
8.0	1.0	1	55.0	1	0	0	1	0
9.0	2.0	1	31.0	1	0	0	1	0
18.0	1.0	1	38.0	1	0	0	1	0
8.0	1.0	1	59.0	1	1	0	0	0
9.0	1.0	1	50.0	1	0	0	1	0
11.0	2.0	1	38.0	1	0	0	1	0
8.0	1.0	1	36.0	2	0	0	0	0
9.0	1.0	2	61.0	1	0	0	0	0
8.0	1.0	1	54.0	1	1	0	0	0
8.0	1.0	1	42.0	1	1	0	0	0
8.0	1.0	1	26.0	1	0	0	0	0
8.0	1.0	1	32.0	2	1	0	0	1
8.0	1.0	1	48.0	1	0	0	1	0
9.0	1.0	1	41.0	1	0	0	0	0
8.0	1.0	1	57.0	1	1	0	1	0
9.0	2.0	1	38.0	1	0	0	1	0
8.0	1.0	1	60.0	1	0	0	1	0
8.0	1.0	1	59.0	2	1	0	1	0
8.0	1.0	2	50.0	1	0	0	1	0
8.0	1.0	1	42.0	1	0	0	0	0
8.0	1.0	1	38.0	1	0	0	1	0
13.0	2.0	1	57.0	1	1	0	0	0
8.0	1.0	1	47.0	1	0	0	1	0
15.0	2.0	2	69.0	2	0	0	1	0
9.0	1.0	1	42.0	1	0	0	0	0
8.0	1.0	2	41.0	2	0	0	0	0
8.0	1.0	1	33.0	1	0	0	1	0
8.0	1.0	1	68.0	1	0	0	1	0
8.0	1.0	1	45.0	2	1	0	0	0
6.0	1.0	1	43.0	2	0	0	0	0
12.0	2.0	1	50.0	2	0	0	0	0
8.0	1.0	1	39.0	2	0	0	1	0
8.0	1.0	1	28.0	1	0	0	0	0
15.0	2.0	1	49.0	1	1	1	1	1
7.0	1.0	2	55.0	1	0	0	1	0
13.0	2.0	1	33.0	2	1	1	1	0
12.0	2.0	1	47.0	2	0	0	0	0
7.0	1.0	2	48.0	2	0	0	1	0
13.0	2.0	2	63.0	2	1	0	1	0
10.0	1.0	1	54.0	1	1	0	0	0
8.0	1.0	1	44.0	1	1	0	1	0
8.0	1.0	1	35.0	2	0	0	1	0

8.0	1.0	1	34.0	1	0	0	0	0
8.0	1.0	1	59.0	1	0	0	1	0
11.0	2.0	1	44.0	2	0	0	1	1
9.0	1.0	1	66.0	2	1	0	1	0
10.0	2.0	2	35.0	1	0	0	0	0
17.0	2.0	1	31.0	2	0	1	0	0
9.0	1.0	1	54.0	1	0	0	1	0
8.0	1.0	1	49.0	1	1	0	1	1
9.0	1.0	2	54.0	1	0	0	0	0
8.0	2.0	2	68.0	2	0	0	1	0
9.0	2.0	1	59.0	2	0	0	1	0
9.0	1.0	1	38.0	1	1	0	0	0
9.0	1.0	1	37.0	1	0	0	1	0
15.0	2.0	1	42.0	2	0	1	0	0
12.0	2.0	1	45.0	2	1	0	1	1
9.0	2.0	1	61.0	2	0	0	0	0
10.0	1.0	1	48.0	1	1	1	0	0
11.0	2.0	1	45.0	1	1	1	1	0
8.0	1.0	1	74.0	2	1	0	1	0
9.0	2.0	1	52.0	2	1	0	1	0
9.0	1.0	1	39.0	1	1	1	0	0
8.0	2.0	1	38.0	2	0	0	1	0
10.0	2.0	1	32.0	1	1	0	1	0
8.0	1.0	1	50.0	2	1	0	1	0
8.0	1.0	1	36.0	1	0	0	0	0
11.0	2.0	1	41.0	1	0	0	1	0
8.0	1.0	1	37.0	1	0	0	1	0
8.0	1.0	1	24.0	2	1	0	0	0
9.0	1.0	1	49.0	1	1	0	1	0
9.0	1.0	1	42.0	1	0	0	1	0
8.0	1.0	1	31.0	1	0	0	1	0
9.0	2.0	1	28.0	1	0	0	1	0
8.0	2.0	1	53.0	2	0	0	1	0
9.0	1.0	1	56.0	1	0	0	1	0
8.0	1.0	1	40.0	1	0	0	0	0
12.0	2.0	1	41.0	1	1	0	1	1
9.0	1.0	1	37.0	1	0	0	1	0
8.0	1.0	1	22.0	1	0	0	1	0
6.0	1.0	1	40.0	2	0	0	1	0
8.0	1.0	1	46.0	2	0	0	1	0
7.0	1.0	1	32.0	2	0	0	0	0
8.0	1.0	1	49.0	1	1	0	0	0
8.0	1.0	1	40.0	1	1	0	0	0
8.0	1.0	1	37.0	1	0	0	1	0
12.0	1.0	1	17.0	1	0	1	0	0
10.0	2.0	1	17.0	1	0	0	0	0

10.0	1.0	1	26.0	1	0	0	1	0
7.0	1.0	1	62.0	1	0	0	1	0
10.0	1.0	1	48.0	1	1	0	1	0
8.0	1.0	1	38.0	1	0	0	1	0
8.0	1.0	1	42.0	1	0	0	1	0
8.0	1.0	1	41.0	1	0	0	0	0
9.0	1.0	1	47.0	2	1	0	1	0
8.0	1.0	2	67.0	2	0	0	1	0
8.0	1.0	1	48.0	1	0	0	1	0
8.0	1.0	1	61.0	1	0	0	0	0
10.0	2.0	1	51.0	1	0	1	1	0
9.0	1.0	1	36.0	1	0	1	0	0
10.0	2.0	1	40.0	2	0	0	1	0
8.0	1.0	1	28.0	1	0	0	0	0
8.0	1.0	1	25.0	2	0	0	0	0
10.0	2.0	1	22.0	1	0	0	0	0
9.0	1.0	2	42.0	2	0	0	1	0
8.0	1.0	1	36.0	1	0	0	0	0
8.0	1.0	1	59.0	2	1	0	1	0
8.0	1.0	1	62.0	2	0	0	1	0
8.0	1.0	1	39.0	1	0	0	0	0
8.0	1.0	2	55.0	1	0	0	1	0
10.0	1.0	1	38.0	1	1	0	1	0
8.0	1.0	1	67.0	1	0	0	1	0
8.0	1.0	1	33.0	1	0	0	1	0
8.0	1.0	2	47.0	1	0	0	0	0
7.0	1.0	1	45.0	1	0	0	0	0
8.0	1.0	1	38.0	1	0	0	0	0
11.0	2.0	2	35.0	1	0	0	1	0
8.0	1.0	1	47.0	1	0	0	1	0
8.0	1.0	1	41.0	1	0	0	1	0
9.0	1.0	1	38.0	1	1	0	1	0
8.0	1.0	1	41.0	1	0	0	0	0
10.0	2.0	1	59.0	2	0	0	1	0
8.0	1.0	1	34.0	1	0	0	0	0
7.0	1.0	1	27.0	2	0	0	0	0
8.0	1.0	1	42.0	1	0	0	0	0
8.0	1.0	1	32.0	1	1	0	0	0
8.0	1.0	1	50.0	1	0	0	1	0
9.0	1.0	1	26.0	1	0	0	1	0
9.0	1.0	1	32.0	1	1	0	0	0
10.0	2.0	1	32.0	1	0	0	1	0
7.0	1.0	1	50.0	2	0	0	1	0
8.0	1.0	1	35.0	1	0	0	1	0
8.0	1.0	1	32.0	1	0	0	0	0
7.0	1.0	1	29.0	1	0	0	0	0

8.0	1.0	1	45.0	1	0	0	1	0
14.0	2.0	1	70.0	2	0	1	0	0
8.0	1.0	1	20.0	1	0	0	1	0
8.0	1.0	1	41.0	1	0	0	1	0
8.0	1.0	1	48.0	2	0	0	1	0
10.0	1.0	2	37.0	1	1	0	1	0
10.0	2.0	1	46.0	1	1	0	1	0
8.0	2.0	2	37.0	1	1	0	1	0
7.0	1.0	1	48.0	2	1	0	1	0
8.0	1.0	1	53.0	1	0	0	1	0
7.0	1.0	2	49.0	1	0	0	0	0
8.0	1.0	1	55.0	1	0	0	0	0
9.0	2.0	1	39.0	2	1	0	1	0
8.0	1.0	1	39.0	1	0	0	1	0
8.0	1.0	1	62.0	1	0	0	1	0
9.0	1.0	1	54.0	1	0	0	1	0
8.0	1.0	1	40.0	1	0	0	1	0
7.0	1.0	2	52.0	1	0	0	1	0
7.0	2.0	1	32.0	1	0	0	0	0
17.0	2.0	1	45.0	1	1	1	1	1
8.0	1.0	1	47.0	1	0	0	1	0
10.0	1.0	1	60.0	1	0	0	0	0
8.0	1.0	1	42.0	1	0	0	1	0
9.0	2.0	1	44.0	2	0	0	0	0
8.0	1.0	1	70.0	2	0	0	0	0
8.0	1.0	1	46.0	1	0	0	1	0
17.0	1.0	2	30.0	1	0	0	1	0
8.0	1.0	1	38.0	2	1	0	1	0
8.0	2.0	1	65.0	2	0	0	1	0
8.0	1.0	1	43.0	1	0	0	0	0
15.0	2.0	1	48.0	1	0	1	1	0
8.0	1.0	1	29.0	1	0	0	1	0
8.0	1.0	1	47.0	1	0	0	0	0
8.0	1.0	1	46.0	1	0	0	1	0
6.0	1.0	1	66.0	1	1	0	0	0
9.0	1.0	1	47.0	1	1	0	0	0
9.0	1.0	1	49.0	1	0	0	0	0
7.0	1.0	1	31.0	2	1	0	0	0
8.0	1.0	1	36.0	1	1	0	0	0
9.0	2.0	1	55.0	2	0	0	1	0
8.0	1.0	1	36.0	2	0	0	1	0
6.0	1.0	2	58.0	2	1	0	1	0
8.0	1.0	1	49.0	1	0	0	1	0
8.0	1.0	1	46.0	1	0	0	1	0
8.0	1.0	2	58.0	1	0	0	1	0
8.0	2.0	1	33.0	1	1	0	1	0

6.0	1.0	1	27.0	2	0	0	1	0
9.0	1.0	1	49.0	1	0	0	1	0
8.0	1.0	2	40.0	1	0	0	1	0
9.0	1.0	1	52.0	2	0	0	1	0
8.0	1.0	1	34.0	1	0	0	1	0
7.0	1.0	1	24.0	1	0	0	1	0
8.0	1.0	1	49.0	1	0	0	1	1
7.0	1.0	1	28.0	2	0	0	1	0
9.0	1.0	1	33.0	1	0	0	1	0
8.0	1.0	1	28.0	1	1	0	1	0
9.0	1.0	1	49.0	1	1	0	0	0
8.0	2.0	1	29.0	1	0	0	0	0
17.0	1.0	1	48.0	1	1	0	1	1
8.0	1.0	1	23.0	1	0	0	1	0
8.0	1.0	1	20.0	1	0	0	1	0
6.0	1.0	2	33.0	2	1	0	0	0
8.0	1.0	1	33.0	1	0	0	1	0
8.0	1.0	1	63.0	2	0	0	0	0
8.0	1.0	1	35.0	1	0	0	1	0
17.0	1.0	1	38.0	1	0	0	1	1
8.0	1.0	1	45.0	1	0	0	0	0
9.0	1.0	1	33.0	2	0	0	1	0
7.0	1.0	1	34.0	1	0	0	0	0
9.0	1.0	1	25.0	1	0	0	1	0
4.0	1.0	1	24.0	2	0	0	0	0
8.0	1.0	1	42.0	1	0	0	1	0
9.0	1.0	1	35.0	1	0	0	0	0
6.0	1.0	1	49.0	1	0	0	1	0
8.0	1.0	1	36.0	1	0	0	1	0
9.0	1.0	1	27.0	1	0	0	1	0
8.0	1.0	1	64.0	1	0	0	1	0
8.0	1.0	1	43.0	1	0	0	1	0
8.0	1.0	1	36.0	1	0	0	1	0
8.0	1.0	1	33.0	1	0	0	1	0
9.0	1.0	1	49.0	2	1	0	1	0
7.0	1.0	1	35.0	1	1	0	0	0
8.0	1.0	1	50.0	1	0	0	1	0
7.0	1.0	1	23.0	2	0	0	0	0
8.0	1.0	1	44.0	1	1	0	0	0
8.0	1.0	1	38.0	1	0	0	1	0
7.0	1.0	1	41.0	2	0	0	1	0
17.0	1.0	1	35.0	1	0	0	0	1
8.0	1.0	1	20.0	1	0	0	0	0
8.0	1.0	1	28.0	2	0	0	0	0
8.0	1.0	1	35.0	2	0	0	1	0
17.0	2.0	1	31.0	2	0	1	0	0

12.0	2.0	1	25.0	1	1	0	0	0
8.0	1.0	1	49.0	1	0	0	1	0
5.0	1.0	1	59.0	2	0	0	0	0
8.0	1.0	1	48.0	1	0	0	0	0
7.0	1.0	1	30.0	1	0	0	1	0
5.0	1.0	1	63.0	2	0	0	0	0
7.0	1.0	1	25.0	1	0	0	0	0
9.0	2.0	2	77.0	2	0	0	1	0
10.0	1.0	1	41.0	1	0	1	1	0
8.0	1.0	1	47.0	1	0	0	1	0
9.0	1.0	1	30.0	1	0	0	1	0
8.0	1.0	1	45.0	1	1	0	0	0
7.0	2.0	2	64.0	2	1	0	1	0
8.0	1.0	1	36.0	1	1	0	0	0
8.0	1.0	1	25.0	1	0	0	1	0
8.0	1.0	1	44.0	1	0	0	1	0
8.0	1.0	1	42.0	1	0	0	0	0
12.0	2.0	1	65.0	2	0	0	0	0
7.0	1.0	1	41.0	1	0	0	1	0
9.0	1.0	2	55.0	1	1	0	0	0
7.0	1.0	1	43.0	2	0	0	1	0
8.0	1.0	1	59.0	1	0	0	1	0
8.0	1.0	1	69.0	1	1	0	0	0
9.0	1.0	2	37.0	2	0	0	0	0
9.0	1.0	1	38.0	1	0	0	1	0
7.0	1.0	1	38.0	2	0	0	0	0
8.0	1.0	1	55.0	2	0	0	1	0
10.0	2.0	1	49.0	1	0	0	0	0
7.0	1.0	1	42.0	1	0	0	1	0
8.0	1.0	1	52.0	2	0	0	0	0
7.0	1.0	1	43.0	1	1	0	0	0
8.0	1.0	1	47.0	1	0	0	0	0
10.0	1.0	1	65.0	1	1	1	1	0
9.0	1.0	1	43.0	1	0	0	1	0
9.0	1.0	1	43.0	1	1	1	1	0
9.0	1.0	1	25.0	1	0	0	0	0
7.0	1.0	1	41.0	2	0	0	1	0
7.0	1.0	1	38.0	1	0	0	1	0
8.0	1.0	1	15.0	1	0	0	0	0
9.0	1.0	1	46.0	1	0	0	1	0
8.0	1.0	1	58.0	1	0	0	1	0
8.0	1.0	1	44.0	2	0	0	0	0
8.0	1.0	1	47.0	2	0	0	1	0
8.0	1.0	1	42.0	1	0	0	1	0
8.0	1.0	1	46.0	1	0	0	0	0
7.0	1.0	1	29.0	1	0	0	0	0

8.0	1.0	1	36.0	1	0	0	1	0
9.0	1.0	1	42.0	1	0	0	1	0
12.0	2.0	1	29.0	1	0	1	1	0
9.0	1.0	1	62.0	2	0	1	1	0
8.0	1.0	1	58.0	2	0	0	1	0
9.0	1.0	1	45.0	2	0	1	0	0
9.0	1.0	1	49.0	1	0	0	1	0
8.0	1.0	1	63.0	1	0	0	0	0
9.0	1.0	1	67.0	1	0	0	0	0
15.0	2.0	1	41.0	2	1	1	1	0
7.0	1.0	1	46.0	1	1	0	1	0
6.0	1.0	1	41.0	2	0	0	1	0
6.0	1.0	1	56.0	2	0	0	0	0
10.0	2.0	1	28.0	1	0	0	1	0
8.0	1.0	1	41.0	2	0	0	0	0
8.0	1.0	1	46.0	1	1	0	1	0
8.0	1.0	1	44.0	1	0	0	1	0
9.0	1.0	1	39.0	1	1	1	1	0
8.0	1.0	2	50.0	2	0	0	0	0
6.0	1.0	2	52.0	2	1	0	1	0
9.0	1.0	1	56.0	1	0	0	1	0
8.0	1.0	1	36.0	2	0	0	1	0
6.0	1.0	1	33.0	2	0	0	0	0
8.0	1.0	1	38.0	1	1	0	0	0
8.0	1.0	1	44.0	1	0	0	1	0
10.0	2.0	1	37.0	1	1	1	0	0
17.0	1.0	1	34.0	1	0	0	1	0
9.0	2.0	1	45.0	2	0	0	0	0
8.0	1.0	1	22.0	1	1	0	0	0
8.0	1.0	1	61.0	1	0	0	1	0
8.0	1.0	2	31.0	1	0	0	0	0
8.0	1.0	2	48.0	1	0	0	1	0
8.0	1.0	1	16.0	1	1	0	0	0
9.0	1.0	1	39.0	1	0	0	1	0
6.0	2.0	2	65.0	2	0	0	1	0
9.0	1.0	1	39.0	1	1	0	0	0
8.0	1.0	2	41.0	1	0	0	0	0
8.0	1.0	1	27.0	1	0	0	1	0
8.0	1.0	1	38.0	1	0	0	1	0
5.0	1.0	1	46.0	2	0	0	1	0
8.0	1.0	1	25.0	1	0	0	1	0
6.0	2.0	2	56.0	2	0	0	1	0
8.0	1.0	1	51.0	1	0	0	1	0
7.0	1.0	1	45.0	2	0	0	0	0
8.0	1.0	1	43.0	1	0	0	0	0
7.0	1.0	1	43.0	2	0	0	0	0

8.0	1.0	1	43.0	2	0	0	0	0
9.0	1.0	1	19.0	1	1	0	0	0
8.0	1.0	1	44.0	1	0	0	0	0
8.0	1.0	1	38.0	1	0	0	1	0
6.0	1.0	1	61.0	2	1	0	1	0
6.0	1.0	1	41.0	2	0	0	0	0
7.0	1.0	1	32.0	2	0	0	1	0
8.0	2.0	1	59.0	2	0	0	1	0
14.0	2.0	1	78.0	2	0	0	0	1

Meld	Bilirubin	Cr	INR	HGB	NLR	**survive time**	Lymphocyte	PLT
39.6	33.4	2.1	3.2	87.0	30.3	8.0	0.5	74.0
41.9	43.5	1.1	6.2	126.0	27.4	12.0	0.9	77.0
28.6	33.6	0.9	2.5	131.0	27.3	14.0	0.8	50.0
30.9	32.4	1.0	2.7	118.0	27.1	11.0	0.6	75.0
52.5	43.4	6.3	3.6	136.0	26.9	8.0	0.5	37.0
25.0	13.7	1.3	1.8	77.0	26.8	8.0	0.8	167.0
37.1	27.6	2.4	2.4	71.0	24.2	14.0	1.2	61.0
22.4	19.1	0.9	1.7	132.0	23.5	23.0	0.4	117.0
18.6	24.2	0.8	1.2	81.0	23.1	22.0	1.2	133.0
37.4	31.4	1.8	3.0	130.0	22.0	6.0	0.7	121.0
23.0	10.7	0.5	3.6	151.0	20.0	19.0	0.7	218.0
39.6	28.5	2.6	2.8	95.0	19.5	7.0	0.5	36.0
17.2	5.8	1.0	1.5	116.0	3.0	11.0	1.2	77.0
21.0	16.3	0.9	1.7	120.0	18.6	18.0	0.4	49.0
14.3	4.0	0.6	2.1	132.0	17.2	27.0	0.9	135.0
43.9	8.2	10.1	1.9	83.0	16.9	9.0	0.8	140.0
20.4	8.3	1.0	1.7	58.0	16.7	5.0	1.0	83.0
30.0	28.5	0.9	3.0	153.0	16.4	21.0	0.3	57.0
32.3	22.6	1.3	2.7	152.0	16.3	9.0	0.6	97.0
28.6	20.3	1.5	1.8	90.0	16.1	8.0	0.3	32.0
29.3	18.4	1.5	2.0	102.0	16.0	2.0	0.4	38.0
28.5	24.4	0.9	2.6	129.0	15.8	21.0	0.5	58.0
27.8	13.7	1.4	2.1	96.0	15.7	5.0	0.7	14.0
42.2	29.7	2.1	4.1	120.0	15.4	7.0	0.5	79.0
35.0	23.6	1.2	3.8	95.0	15.4	3.0	0.8	229.0
20.5	24.9	0.8	1.4	130.0	15.4	30.0	4.0	172.0
14.7	4.1	0.8	1.6	128.0	15.0	30.0	1.6	37.0
30.8	18.1	1.0	3.3	134.0	14.9	16.0	0.2	176.0
23.3	6.8	0.8	2.8	150.0	14.9	6.0	1.1	48.0
42.0	36.0	2.5	3.2	123.0	14.7	4.0	0.7	109.0
45.0	43.8	3.9	2.8	108.0	14.6	2.0	0.6	17.0
30.6	19.5	0.9	3.5	138.0	14.4	4.0	1.2	278.0
35.9	28.5	1.1	4.2	147.0	14.3	21.0	1.4	66.0
16.3	4.7	1.1	1.3	79.0	14.2	22.0	1.5	145.0
30.6	48.3	1.1	2.2	101.0	14.2	16.0	1.0	68.0
23.6	27.8	0.7	2.1	142.0	13.9	30.0	0.7	126.0
30.1	16.0	1.1	3.1	139.0	13.9	19.0	1.9	188.0
27.9	34.7	0.9	2.4	139.0	13.9	3.0	0.6	101.0
63.4	22.6	1.0	56.7	136.0	13.8	6.0	1.1	46.0
37.1	31.2	2.7	2.1	136.0	13.8	6.0	1.1	46.0
33.1	15.5	1.0	4.2	98.0	13.8	9.0	1.2	112.0
31.4	22.5	0.9	3.7	134.0	13.8	5.0	0.5	111.0
47.4	22.5	5.6	3.1	99.0	13.5	21.0	1.9	105.0
25.0	31.7	1.0	1.7	135.0	13.4	18.0	0.8	59.0
23.5	21.8	0.8	2.0	105.0	13.2	30.0	0.4	111.0

28.8	22.1	1.4	2.0	142.0	13.1	15.0	0.8	402.0
38.7	38.2	2.5	2.4	130.0	12.9	30.0	0.7	232.0
37.4	4.1	4.9	2.6	55.0	12.7	6.0	0.8	28.0
31.1	34.1	1.3	2.3	133.0	12.7	15.0	0.5	89.0
30.3	34.2	0.7	3.4	125.0	12.6	10.0	0.7	103.0
25.9	23.9	1.0	2.0	130.0	12.6	15.0	0.8	123.0
24.1	27.7	1.1	1.5	121.0	12.4	13.0	2.3	106.0
32.6	24.6	1.3	2.8	117.0	12.2	7.0	0.9	52.0
37.4	33.4	1.6	3.2	175.0	12.2	2.0	1.0	132.0
30.0	22.3	1.7	1.8	101.0	12.1	30.0	0.7	79.0
32.7	26.9	2.1	1.8	87.0	12.1	10.0	2.0	58.0
35.2	47.1	1.9	2.1	110.0	12.0	13.0	1.2	107.0
33.9	29.5	1.5	2.7	87.0	11.8	4.0	1.5	91.0
23.2	0.5	4.0	1.7	54.0	11.7	10.0	0.5	45.0
21.8	6.0	1.1	1.9	99.0	11.6	26.0	0.7	63.0
25.1	24.2	0.9	1.9	134.0	11.5	30.0	3.0	169.0
35.6	28.2	2.4	2.1	113.0	11.5	5.0	0.9	84.0
23.2	16.4	0.7	2.2	143.0	11.5	13.0	0.5	65.0
45.6	33.4	3.0	4.0	156.0	11.5	9.0	1.8	112.0
28.7	29.7	0.9	2.6	164.0	11.5	7.0	0.4	54.0
21.4	20.8	0.9	1.5	147.0	11.5	30.0	0.5	31.0
59.7	58.8	6.9	5.6	78.0	11.4	2.0	0.9	86.0
28.5	28.2	1.3	1.9	134.0	11.3	30.0	1.1	114.0
36.8	23.3	2.2	2.7	51.0	11.3	13.0	0.5	50.0
24.1	27.3	0.9	1.7	138.0	11.2	30.0	0.7	89.0
17.0	11.2	0.8	1.5	96.0	11.2	30.0	0.8	107.0
19.1	21.2	0.6	1.7	90.0	11.2	16.0	0.4	128.0
26.0	23.1	0.7	2.7	100.0	11.1	19.0	0.8	1.0
28.2	28.3	0.8	2.8	123.0	11.0	10.0	0.9	61.0
43.8	37.6	2.7	3.5	82.0	11.0	2.0	2.0	123.0
18.0	13.2	1.1	1.1	95.0	11.0	23.0	0.5	169.0
38.7	21.1	1.1	6.0	105.0	11.0	3.0	0.6	56.0
24.1	3.8	1.1	2.9	144.0	10.8	8.0	0.9	114.0
35.7	14.7	3.2	2.0	116.0	10.6	9.0	0.7	122.0
27.8	31.2	0.9	2.4	125.0	10.5	4.0	0.8	48.0
23.0	18.4	1.0	1.6	151.0	10.3	30.0	1.6	79.0
24.8	23.4	0.9	1.9	137.0	10.3	30.0	0.7	79.0
35.6	16.6	0.9	5.8	148.0	10.2	5.0	0.4	108.0
13.7	10.1	0.8	1.1	123.0	10.2	29.0	0.5	172.0
35.1	35.3	1.8	2.4	111.0	10.2	22.0	0.3	128.0
20.3	11.4	0.8	1.8	124.0	10.1	30.0	1.5	91.0
22.9	21.3	0.8	1.9	137.0	10.1	20.0	0.7	77.0
33.1	31.6	1.1	3.1	147.0	10.0	14.0	1.1	106.0
26.7	21.9	1.1	2.0	119.0	10.0	5.0	1.1	136.0
19.5	6.0	0.7	2.3	128.0	9.8	19.0	0.8	62.0
38.9	27.3	4.0	1.8	144.0	9.8	24.0	0.4	72.0

33.0	27.2	1.8	2.1	98.0	9.6	10.0	0.9	33.0
28.3	21.7	1.1	2.3	106.0	9.4	22.0	0.9	90.0
50.7	24.3	0.9	19.4	91.0	9.3	16.0	0.9	162.0
43.7	42.2	2.6	3.5	139.0	9.3	10.0	0.5	98.0
30.7	21.9	1.5	2.2	75.0	9.3	29.0	0.3	13.0
19.3	14.8	0.6	1.9	124.0	9.3	30.0	0.4	33.0
34.4	22.3	2.6	1.8	150.0	9.1	30.0	2.1	107.0
27.1	36.2	0.8	2.2	153.0	9.0	19.0	1.5	99.0
26.5	35.2	0.6	2.7	107.0	9.0	3.0	0.9	9.3
20.5	15.8	0.8	1.7	135.0	8.9	30.0	1.5	58.0
38.0	32.3	2.6	2.3	132.0	8.9	2.0	0.9	93.0
23.1	16.9	1.0	1.8	125.0	8.9	30.0	0.8	80.0
22.3	12.7	0.8	2.2	103.0	8.9	6.0	1.1	42.0
32.0	29.0	0.9	3.5	120.0	8.9	15.0	0.4	119.0
24.5	28.4	0.9	1.8	116.0	8.8	30.0	1.8	41.0
21.8	23.4	0.8	1.6	144.0	8.8	29.0	0.7	153.0
23.9	27.7	0.9	1.7	108.0	8.7	30.0	1.7	80.0
24.8	28.9	1.0	1.7	110.0	8.7	30.0	0.8	87.0
27.6	36.8	0.8	2.3	116.0	8.7	30.0	1.9	90.0
29.7	19.1	0.7	4.1	148.0	8.6	28.0	1.0	120.0
31.8	31.3	1.0	3.0	171.0	8.6	30.0	0.6	91.0
25.2	23.9	0.6	2.8	104.0	8.6	12.0	0.8	58.0
28.7	26.9	1.2	2.0	139.0	8.5	30.0	0.9	117.0
35.7	38.6	1.4	2.9	152.0	8.4	30.0	1.1	130.0
31.2	27.0	1.6	2.0	116.0	8.2	30.0	1.0	88.0
30.0	31.5	2.2	1.3	136.0	8.2	5.0	0.7	138.0
23.9	25.6	0.9	1.8	59.0	8.2	25.0	2.6	276.0
45.2	12.6	0.6	21.3	125.0	8.2	30.0	1.9	50.0
26.5	29.6	0.9	2.1	133.0	8.1	30.0	0.7	107.0
29.3	36.1	1.4	1.7	127.0	8.1	9.0	0.8	72.0
41.5	13.8	1.1	8.5	161.0	8.1	3.0	1.5	59.0
37.0	38.4	1.9	2.5	122.0	8.0	15.0	0.5	88.0
32.4	13.7	1.8	2.6	128.0	8.0	30.0	1.8	165.0
37.9	28.3	1.5	3.7	76.0	8.0	3.0	0.7	155.0
43.3	47.8	4.1	2.2	112.0	7.9	8.0	2.3	47.0
29.2	29.9	0.7	3.3	109.0	7.8	4.0	1.4	212.0
22.5	14.4	0.7	2.3	166.0	7.8	5.0	0.7	110.0
21.4	15.1	0.7	2.0	139.0	7.8	30.0	2.3	107.0
22.8	24.7	0.8	1.8	148.0	7.7	11.0	1.6	146.0
32.1	24.0	0.7	4.5	129.0	7.7	2.0	2.3	117.0
25.3	12.5	0.9	2.5	122.0	7.6	30.0	1.4	27.0
19.9	14.8	0.6	2.0	128.0	7.5	9.0	1.3	61.0
10.6	23.6	0.4	1.1	97.0	7.5	30.0	0.5	42.0
42.7	27.8	1.6	5.6	109.0	7.5	5.0	2.3	111.0
33.8	11.5	2.4	2.4	65.0	7.5	11.0	0.7	23.0
28.2	25.8	0.7	3.0	122.0	7.4	30.0	0.8	88.0

31.2	24.8	1.8	1.8	108.0	7.4	10.0	0.6	44.0
29.4	33.4	1.2	2.0	199.0	7.3	30.0	1.2	125.0
30.9	35.6	0.9	2.8	116.0	7.2	12.0	1.2	78.0
26.6	27.3	0.9	2.3	120.0	7.2	28.0	1.2	68.0
30.2	40.6	1.1	2.2	117.0	7.2	6.0	1.1	40.0
18.3	9.7	0.9	1.4	146.0	7.2	30.0	1.0	144.0
25.6	12.6	0.7	3.1	138.0	7.1	30.0	1.3	87.0
20.2	7.8	0.7	2.3	110.0	7.0	30.0	1.9	49.0
13.5	8.4	0.5	1.6	84.0	7.0	30.0	0.5	37.0
21.5	7.0	1.0	2.0	82.0	7.0	3.0	1.3	44.0
27.0	26.6	1.0	2.1	123.0	6.9	11.0	1.6	99.0
25.8	18.5	0.9	2.4	132.0	6.8	24.0	0.9	60.0
28.6	29.7	0.9	2.5	152.0	6.7	30.0	1.4	93.0
29.0	42.9	0.8	2.7	122.0	6.7	4.0	1.8	140.0
35.4	14.8	1.9	3.1	132.0	6.7	2.0	1.4	71.0
20.2	1.9	0.9	3.0	149.0	6.7	30.0	1.2	118.0
30.7	21.7	1.2	2.7	85.0	6.6	24.0	0.9	36.0
20.4	15.1	1.0	1.5	108.0	6.6	19.0	0.8	155.0
19.2	20.5	0.8	1.4	97.0	6.6	18.0	2.4	162.0
28.1	11.5	0.8	3.6	139.0	6.6	8.0	0.4	80.0
28.8	23.3	0.7	3.5	95.0	6.6	30.0	0.6	60.0
21.8	17.6	0.9	1.7	138.0	6.6	30.0	1.7	170.0
30.5	23.5	1.3	2.3	109.0	6.6	30.0	2.0	109.0
48.3	35.5	7.5	2.3	91.0	6.6	6.0	1.2	69.0
21.6	25.4	0.9	1.5	149.0	6.5	30.0	1.4	101.0
39.3	21.6	2.5	3.0	109.0	6.5	30.0	1.1	92.0
24.3	9.6	0.9	2.5	183.0	6.3	8.0	1.4	190.0
28.9	35.1	1.1	2.1	152.0	6.3	6.0	1.9	64.0
44.6	17.6	4.7	3.1	130.0	6.3	3.0	4.2	109.0
29.7	21.0	1.7	1.8	118.0	6.0	30.0	0.5	23.0
21.8	25.4	0.9	1.5	125.0	6.2	30.0	1.3	107.0
31.9	15.1	1.7	2.5	144.0	6.2	30.0	1.3	217.0
19.9	9.0	0.9	1.7	50.0	6.2	1.0	2.0	27.0
29.5	25.6	1.0	2.6	172.0	6.2	30.0	2.1	113.0
22.1	13.0	0.7	2.2	132.0	6.2	30.0	1.2	53.0
36.0	21.5	1.5	3.5	84.0	6.2	6.0	1.6	43.0
22.1	15.7	0.6	2.4	79.0	6.2	9.0	0.4	30.0
29.9	32.8	0.8	3.1	134.0	6.1	5.0	1.5	27.0
15.4	1.6	1.3	1.5	72.0	6.1	30.0	0.7	116.0
22.8	23.5	0.9	1.7	142.0	6.1	30.0	0.4	53.0
26.6	18.3	1.0	2.4	128.0	6.0	5.0	1.2	131.0
14.9	4.6	0.9	1.3	173.0	6.0	30.0	1.4	64.0
29.7	35.7	0.7	3.4	137.0	6.0	8.0	1.0	180.0
21.0	28.0	0.8	1.4	132.0	5.9	30.0	0.3	32.0
12.5	19.2	0.5	1.1	110.0	5.9	30.0	1.4	212.0
18.4	16.1	0.8	1.4	81.0	5.9	30.0	0.4	11.0

26.8	18.8	0.7	3.1	142.0	5.8	10.0	1.7	73.0
17.7	5.9	0.9	1.6	140.0	5.8	30.0	1.9	111.0
24.8	13.3	0.9	2.3	143.0	5.8	23.0	0.8	49.0
26.2	27.8	0.7	2.7	151.0	5.7	4.0	1.0	97.0
20.2	7.5	1.1	1.6	125.0	5.7	6.0	1.6	89.0
24.4	27.0	0.6	2.6	88.0	5.7	9.0	0.7	94.0
26.9	14.5	1.1	2.3	129.0	5.7	11.0	1.1	78.0
25.7	25.1	1.0	1.9	105.0	5.6	9.0	1.3	108.0
27.5	22.6	0.7	3.1	151.0	5.6	30.0	1.9	139.0
32.4	21.9	1.5	2.5	142.0	5.6	12.0	1.4	241.0
33.1	16.7	1.8	2.5	101.0	5.6	30.0	1.7	39.0
34.8	15.4	1.7	3.2	133.0	5.5	10.0	1.6	74.0
25.9	27.7	0.7	2.4	138.0	5.5	30.0	1.5	124.0
30.3	31.4	0.7	3.6	140.0	5.5	5.0	1.5	91.0
28.5	38.0	0.7	2.9	123.0	5.4	30.0	1.5	76.0
18.0	12.5	0.6	2.0	102.0	5.4	30.0	2.1	63.0
26.9	14.9	1.0	2.6	150.0	5.4	30.0	1.2	170.0
29.0	39.8	1.6	1.5	113.0	5.4	30.0	0.7	187.0
20.6	32.1	0.6	1.8	138.0	5.4	14.0	1.0	40.0
29.5	35.4	1.2	2.0	146.0	5.4	5.0	1.9	64.0
21.2	21.1	0.8	1.6	158.0	5.3	30.0	1.6	117.0
24.5	39.6	1.0	1.5	133.0	5.3	30.0	0.8	89.0
24.3	19.7	0.7	2.4	125.0	5.3	9.0	1.3	68.0
28.3	17.3	1.2	2.4	92.0	5.3	6.0	2.3	88.0
24.4	19.7	1.3	1.5	85.0	5.2	13.0	0.9	52.0
15.3	7.1	0.9	1.3	86.0	5.2	30.0	0.3	40.0
12.0	3.2	0.5	2.0	136.0	5.2	15.0	1.5	124.0
26.4	25.8	1.0	2.0	110.0	5.2	16.0	2.4	93.0
25.4	26.5	0.9	2.0	125.0	5.1	19.0	1.4	118.0
28.6	33.0	1.4	1.7	149.0	5.1	17.0	1.1	101.0
22.8	22.6	0.9	1.6	96.0	5.1	25.0	0.9	80.0
32.4	26.7	1.1	3.2	115.0	5.1	30.0	1.9	78.0
19.6	12.4	0.8	1.7	145.0	5.0	30.0	1.1	78.0
29.9	25.2	0.8	3.3	166.0	5.0	21.0	2.2	88.0
44.0	30.5	1.8	5.5	144.0	5.0	4.0	2.3	256.0
22.5	19.8	0.7	2.1	134.0	4.9	30.0	1.1	34.0
28.4	39.1	1.6	1.4	120.0	4.9	30.0	0.8	85.0
28.8	26.9	0.8	2.9	80.0	4.8	12.0	1.2	191.0
37.7	25.1	1.2	4.7	113.0	4.8	5.0	1.5	72.0
28.6	20.0	1.1	2.5	121.0	4.8	30.0	1.1	101.0
25.3	19.5	1.9	1.2	188.0	4.8	12.0	0.9	84.0
27.9	18.0	1.0	2.6	112.0	4.8	29.0	1.3	45.0
21.7	20.8	0.8	1.8	95.0	4.7	30.0	0.8	98.0
26.4	37.0	0.9	1.9	95.0	4.7	30.0	1.5	65.0
28.3	23.9	1.1	2.2	145.0	4.7	30.0	1.1	105.0
24.1	27.2	1.1	1.4	129.0	4.7	14.0	1.2	89.0

20.0	25.2	0.8	1.5	85.0	4.7	30.0	0.7	167.0
23.8	23.0	1.0	1.6	125.0	4.7	30.0	1.3	116.0
29.9	15.9	1.3	2.6	102.0	4.7	30.0	1.9	23.0
13.3	6.3	0.5	1.8	112.0	4.7	30.0	3.0	96.0
26.9	49.8	1.1	1.6	102.0	4.6	30.0	2.0	60.0
14.5	2.2	0.8	1.9	146.0	4.6	15.0	2.6	91.0
35.5	27.5	1.8	2.6	123.0	4.6	30.0	1.3	124.0
20.2	12.0	0.7	2.0	105.0	4.5	30.0	0.6	87.0
26.4	21.1	0.8	2.6	147.0	4.5	30.0	1.8	119.0
23.8	17.1	0.9	2.0	141.0	4.5	11.0	0.8	129.0
23.3	16.2	0.9	1.9	140.0	4.5	30.0	0.8	113.0
26.7	23.9	0.7	2.7	140.0	4.4	30.0	1.2	135.0
22.3	31.5	0.7	1.7	135.0	4.4	30.0	1.1	94.0
23.6	24.6	1.0	1.6	124.0	4.4	30.0	1.2	178.0
18.9	18.6	0.8	1.3	152.0	4.4	30.0	1.0	90.0
29.0	29.2	1.9	1.4	164.0	4.4	30.0	2.0	123.0
25.8	19.9	0.9	2.2	145.0	4.4	30.0	0.6	116.0
25.8	25.1	0.6	2.8	116.0	4.4	30.0	2.0	162.0
21.1	30.2	0.9	1.3	101.0	4.4	30.0	1.3	212.0
30.7	29.5	1.1	2.5	140.0	4.4	30.0	1.0	75.0
22.0	16.5	0.7	2.2	116.0	4.4	30.0	1.2	115.0
24.1	14.1	0.9	2.1	100.0	4.4	30.0	1.1	45.0
18.0	15.4	0.7	1.6	120.0	4.4	30.0	0.2	52.0
25.4	32.1	0.9	1.8	144.0	4.3	30.0	1.2	110.0
26.5	28.4	0.9	2.0	151.0	4.3	30.0	1.3	95.0
40.7	43.3	1.4	4.5	81.0	4.3	10.0	0.8	35.0
24.8	19.8	1.0	1.9	125.0	4.3	30.0	0.5	82.0
37.3	23.2	1.5	4.0	145.0	4.3	3.0	2.2	318.0
26.6	16.8	0.8	2.8	123.0	4.3	30.0	2.0	123.0
17.0	15.7	0.5	1.7	90.0	4.3	30.0	0.6	131.0
20.7	17.0	1.0	1.4	160.0	4.3	30.0	1.4	166.0
22.4	15.5	1.1	1.5	121.0	4.3	30.0	1.1	116.0
23.7	19.7	0.9	1.8	156.0	4.3	30.0	1.5	147.0
13.5	13.4	0.7	1.1	133.0	4.2	30.0	1.3	7.1
27.4	16.5	0.6	3.7	141.0	4.2	4.0	1.1	110.0
21.9	13.9	0.7	2.3	101.0	4.2	30.0	0.9	25.0
21.3	14.5	0.8	1.9	155.0	4.2	30.0	2.2	167.0
32.8	32.0	1.7	2.0	123.0	4.2	8.0	1.0	267.0
20.9	10.0	0.8	2.1	104.0	4.2	30.0	1.1	59.0
31.7	25.6	0.9	3.5	95.0	4.2	7.0	1.3	84.0
29.9	25.1	0.8	3.3	158.0	4.2	5.0	0.9	149.0
22.6	27.8	1.1	1.3	114.0	4.2	30.0	1.5	254.0
32.7	27.4	0.8	4.1	125.0	4.2	2.0	1.8	128.0
33.6	24.3	2.4	1.8	131.0	4.2	30.0	1.5	119.0
20.1	17.5	0.9	1.4	112.0	4.1	30.0	0.6	54.0
22.7	22.8	1.0	1.5	144.0	4.1	30.0	0.9	181.0

18.8	17.1	0.7	1.5	151.0	4.1	30.0	0.8	222.0
23.6	8.5	0.7	3.0	92.0	4.1	30.0	0.6	39.0
39.4	28.1	3.1	2.4	91.0	4.1	13.0	0.5	22.0
36.0	27.4	1.2	4.0	93.0	4.1	30.0	1.9	127.0
29.7	25.1	1.0	2.7	121.0	4.1	25.0	0.7	77.0
34.0	15.3	1.3	3.8	125.0	4.1	3.0	1.7	80.0
28.3	23.2	0.8	3.0	139.0	4.0	30.0	0.4	62.0
26.0	30.5	0.8	2.2	106.0	4.0	30.0	2.3	69.0
25.4	23.0	1.4	1.4	131.0	4.0	30.0	1.6	144.0
25.5	16.4	1.0	2.2	74.0	4.0	23.0	0.4	40.0
27.7	14.1	1.5	2.0	133.0	4.0	22.0	1.8	78.0
27.8	27.1	1.3	1.8	94.0	4.0	30.0	0.9	117.0
39.3	12.2	0.8	9.7	151.0	4.0	30.0	1.6	107.0
33.4	21.9	0.8	5.0	126.0	4.0	4.0	1.6	141.0
39.8	23.5	3.7	2.2	96.0	4.0	9.0	1.4	85.0
17.2	13.6	0.5	1.9	123.0	4.0	15.0	1.0	144.0
18.8	23.0	0.8	1.3	122.0	4.0	30.0	0.9	159.0
28.2	20.5	1.1	2.3	126.0	4.0	23.0	1.2	68.0
25.9	19.5	1.1	1.9	106.0	4.0	30.0	1.6	41.0
19.8	16.2	0.6	1.9	128.0	4.0	8.0	0.7	28.0
20.6	30.8	0.5	2.0	135.0	4.0	30.0	1.1	93.0
27.4	27.9	1.5	1.5	116.0	4.0	3.0	2.2	109.0
35.4	35.4	0.8	4.9	136.0	4.0	27.0	1.5	157.0
21.8	20.2	0.9	1.6	128.0	4.0	30.0	1.5	106.0
22.9	28.9	1.0	1.4	124.0	3.9	30.0	0.9	163.0
24.8	20.7	0.8	2.2	121.0	3.9	12.0	2.7	107.0
26.4	44.1	1.1	1.5	102.0	3.9	30.0	0.7	106.0
25.8	19.4	0.9	2.3	170.0	3.9	30.0	1.1	99.0
24.8	12.6	1.0	2.1	94.0	3.9	30.0	0.3	79.0
50.6	22.4	0.9	19.5	118.0	3.9	30.0	2.0	78.0
19.5	28.6	0.6	1.7	127.0	3.9	30.0	0.4	53.0
20.9	12.1	0.8	1.9	140.0	3.9	30.0	1.0	210.0
26.7	22.7	0.9	2.4	136.0	3.9	21.0	1.5	156.0
19.8	14.1	0.7	1.9	100.0	3.9	30.0	0.8	68.0
24.4	19.5	0.8	2.2	130.0	3.9	30.0	1.1	157.0
32.0	29.6	1.7	2.0	102.0	3.8	13.0	2.0	91.0
26.5	22.1	0.8	2.6	120.0	3.8	30.0	1.0	60.0
24.1	31.9	1.0	1.5	165.0	3.8	30.0	1.6	192.0
18.1	14.5	1.0	1.2	140.0	3.8	30.0	0.8	83.0
26.7	28.9	1.0	1.9	139.0	3.8	30.0	1.2	85.0
16.7	11.5	0.7	1.6	111.0	3.8	30.0	2.3	108.0
23.1	13.8	0.8	2.1	127.0	3.8	30.0	1.8	42.0
21.1	20.1	0.6	2.0	116.0	3.8	30.0	1.1	99.0
22.9	27.9	0.7	1.9	133.0	3.8	30.0	1.0	80.0
28.6	46.4	0.7	2.8	143.0	3.8	30.0	2.6	47.0
28.6	46.4	0.7	2.8	143.0	3.8	10.0	2.6	47.0

27.9	33.9	1.3	1.7	96.0	3.7	30.0	1.0	65.0
17.0	10.0	0.9	1.3	112.0	3.7	30.0	0.6	39.0
33.0	18.5	1.4	2.9	123.0	3.7	30.0	1.1	90.0
24.9	22.8	1.0	1.8	129.0	3.7	30.0	1.3	159.0
24.0	35.4	0.9	1.6	121.0	3.7	30.0	1.4	219.0
22.9	17.3	0.7	2.1	122.0	3.7	30.0	0.7	150.0
32.2	33.4	1.1	2.9	96.0	3.7	30.0	2.7	93.0
23.9	12.1	1.6	1.4	118.0	3.7	30.0	1.8	74.0
26.4	20.2	0.9	2.3	125.0	3.7	30.0	1.5	116.0
24.8	19.1	1.1	1.8	173.0	3.7	30.0	1.9	150.0
24.1	14.3	0.9	2.2	116.0	3.7	18.0	0.7	53.0
23.9	33.5	0.8	1.7	124.0	3.7	30.0	1.0	78.0
37.0	15.5	1.1	5.4	149.0	3.7	7.0	1.5	68.0
21.6	13.7	0.8	2.0	141.0	3.6	30.0	2.1	140.0
22.8	20.0	0.9	1.7	126.0	3.6	30.0	1.3	209.0
18.0	28.0	0.7	1.2	127.0	3.6	17.0	1.4	340.0
28.8	15.8	0.9	3.2	78.0	3.6	30.0	1.8	97.0
27.3	20.9	1.1	2.2	149.0	3.6	30.0	1.1	208.0
24.0	25.1	0.9	1.8	80.0	3.6	30.0	0.9	58.0
19.3	12.1	0.6	2.2	107.0	3.6	30.0	0.4	30.0
30.1	4.5	0.7	6.4	147.0	3.6	30.0	0.7	77.0
20.6	17.3	0.5	2.3	108.0	3.6	30.0	1.4	69.0
30.4	20.0	1.3	2.5	104.0	3.5	30.0	0.9	42.0
25.3	20.5	0.9	2.1	118.0	3.5	30.0	1.6	103.0
24.7	26.0	1.1	1.6	138.0	3.5	30.0	2.1	100.0
21.6	14.8	0.7	2.0	124.0	3.5	30.0	1.0	224.0
21.8	8.2	1.2	1.7	139.0	3.5	30.0	1.2	150.0
23.3	20.3	0.8	1.9	118.0	3.5	30.0	1.5	143.0
19.3	23.7	0.6	1.6	108.0	3.5	13.0	1.0	49.0
21.5	24.7	0.9	1.4	154.0	3.5	30.0	1.9	159.0
24.6	30.4	1.1	1.5	106.0	3.5	30.0	0.9	85.0
21.4	13.9	0.7	2.2	94.0	3.5	30.0	0.8	54.0
23.2	21.3	0.8	1.9	152.0	3.5	30.0	1.0	230.0
31.0	24.2	1.1	2.9	120.0	3.5	9.0	2.2	133.0
24.0	19.8	0.8	2.0	161.0	3.5	30.0	1.9	88.0
21.3	19.4	1.0	1.4	153.0	3.5	30.0	2.0	76.0
24.1	16.5	1.1	1.8	140.0	3.5	30.0	0.9	145.0
21.3	25.4	0.8	1.5	129.0	3.4	30.0	1.5	73.0
25.7	20.1	1.1	1.9	131.0	3.4	30.0	1.3	108.0
33.0	40.1	1.1	2.8	116.0	3.4	30.0	1.2	96.0
24.0	35.9	1.0	1.5	75.0	3.4	30.0	1.0	94.0
32.7	25.2	1.1	3.3	155.0	3.4	30.0	0.8	105.0
19.9	7.5	0.8	2.1	155.0	3.4	30.0	1.3	139.0
23.5	16.5	0.9	1.9	135.0	3.4	30.0	1.5	103.0
15.5	12.9	0.8	1.1	136.0	3.4	30.0	1.6	187.0
21.1	10.8	0.8	1.9	159.0	3.4	30.0	0.9	140.0

20.2	24.6	0.7	1.5	102.0	3.4	30.0	0.9	90.0
28.5	15.8	1.2	2.4	177.0	3.4	4.0	2.1	81.0
21.2	22.6	0.6	2.1	125.0	3.7	30.0	1.4	96.0
23.4	24.2	0.8	1.8	129.0	3.7	30.0	0.8	130.0
23.8	20.6	1.0	1.7	90.0	3.5	30.0	1.0	138.0
28.5	23.1	1.4	1.9	96.0	3.5	30.0	0.8	56.0
31.5	29.6	1.1	2.8	164.0	3.4	26.0	2.0	159.0
28.5	23.1	1.4	1.9	96.0	3.3	18.0	0.8	56.0
18.0	7.5	0.9	1.6	105.0	3.5	30.0	1.0	40.0
28.3	28.7	1.0	2.4	112.0	3.3	30.0	1.2	55.0
19.6	6.6	1.0	1.7	135.0	3.3	30.0	1.9	332.0
19.1	17.2	0.9	1.3	131.0	3.3	30.0	0.9	121.0
24.2	18.6	0.6	2.8	80.0	3.3	25.0	0.4	45.0
24.5	31.3	0.9	1.7	138.0	3.3	30.0	1.1	56.0
25.9	23.6	0.8	2.3	105.0	3.3	30.0	1.3	115.0
28.6	20.7	1.0	2.6	121.0	3.5	30.0	1.0	39.0
19.0	6.6	0.7	2.1	163.0	3.3	30.0	0.7	71.0
24.4	11.6	1.0	2.2	112.0	3.5	30.0	0.8	33.0
20.9	35.9	1.2	1.0	141.0	3.3	20.0	1.8	291.0
37.1	26.3	2.1	2.7	147.0	3.1	13.0	1.0	157.0
23.8	32.6	0.9	1.6	126.0	3.0	30.0	0.5	93.0
31.8	22.3	1.9	1.9	99.0	3.0	30.0	0.3	54.0
20.2	8.7	1.4	1.3	141.0	3.0	30.0	2.2	177.0
69.3	32.3	1.1	79.5	139.0	3.0	24.0	1.7	78.0
19.9	12.6	0.6	2.2	135.0	3.0	30.0	1.1	95.0
19.5	22.8	0.6	1.7	127.0	3.0	30.0	1.4	184.0
31.0	20.3	0.9	3.5	111.0	3.0	30.0	0.5	106.0
27.3	27.9	0.9	2.4	124.0	3.0	30.0	1.6	248.0
26.0	11.4	1.2	2.2	124.0	3.1	18.0	1.7	163.0
20.0	6.4	0.9	2.0	109.0	3.1	30.0	0.7	11.0
36.3	40.6	2.0	2.2	97.0	3.1	22.0	1.2	38.0
22.5	26.0	0.9	1.5	125.0	3.1	30.0	1.0	92.0
26.9	14.4	1.1	2.4	125.0	3.1	30.0	1.5	96.0
27.4	26.6	1.0	2.2	82.0	3.1	30.0	1.3	108.0
16.9	3.2	0.9	1.8	163.0	3.0	30.0	1.3	151.0
23.3	13.1	0.8	2.3	108.0	3.0	30.0	0.9	57.0
27.2	15.5	0.8	3.0	117.0	3.0	30.0	1.1	196.0
19.7	22.2	0.9	1.3	160.0	3.0	30.0	1.3	152.0
26.3	25.6	1.0	2.0	150.0	3.0	30.0	1.9	110.0
15.5	7.5	0.8	1.4	98.0	3.0	4.0	0.8	73.0
27.7	27.5	1.1	2.0	100.0	3.0	30.0	1.1	57.0
15.4	15.8	0.7	1.2	153.0	3.0	30.0	1.1	153.0
27.8	35.5	0.9	2.2	115.0	3.0	30.0	1.6	39.0
20.3	16.3	0.9	1.5	161.0	3.0	30.0	1.8	225.0
25.6	23.0	0.8	2.4	127.0	3.0	30.0	0.6	142.0
28.5	30.1	1.3	1.8	162.0	3.0	30.0	1.6	106.0

12.5	4.7	0.5	2.0	50.0	3.0	30.0	0.3	28.0
47.4	21.0	0.7	19.6	122.0	2.9	30.0	0.6	80.0
22.0	24.6	0.6	2.0	131.0	2.9	30.0	0.9	78.0
24.2	13.0	1.2	1.7	68.0	2.9	30.0	1.3	55.0
23.2	37.7	0.9	1.5	122.0	2.9	30.0	2.0	155.0
20.1	8.7	0.7	2.2	150.0	2.9	30.0	1.1	118.0
28.0	33.8	1.1	2.0	156.0	2.9	30.0	1.2	116.0
17.9	20.9	0.8	1.3	84.0	2.9	30.0	0.4	141.0
31.7	41.6	1.7	1.8	126.0	2.8	30.0	1.0	80.0
24.4	31.7	1.0	1.5	122.0	2.8	30.0	1.7	85.0
23.7	17.3	0.8	2.1	119.0	2.8	30.0	2.5	99.0
20.6	9.7	1.0	1.7	147.0	2.8	21.0	0.7	62.0
38.3	28.7	1.6	3.7	105.0	2.8	30.0	1.3	70.0
22.3	20.3	0.6	2.2	129.0	2.8	30.0	1.4	105.0
26.5	19.7	0.9	2.4	136.0	2.8	30.0	0.9	64.0
20.0	18.9	1.1	1.1	107.0	2.8	30.0	1.9	119.0
24.4	17.0	0.8	2.3	165.0	2.8	30.0	1.3	144.0
26.5	30.7	1.6	1.3	114.0	2.7	30.0	1.7	122.0
21.0	16.3	1.0	1.5	162.0	2.7	30.0	1.8	255.0
33.8	45.0	1.9	1.8	130.0	2.7	30.0	0.8	104.0
24.9	23.6	0.8	2.1	143.0	2.7	30.0	1.1	70.0
23.0	20.4	0.9	1.8	123.0	2.7	30.0	1.0	96.0
20.9	11.1	1.0	1.6	164.0	2.7	30.0	1.4	88.0
23.2	17.1	0.9	1.9	127.0	2.7	30.0	1.3	106.0
12.1	4.8	0.9	1.1	160.0	2.7	30.0	0.9	87.0
22.9	10.6	1.3	1.5	138.0	2.7	30.0	1.0	96.0
29.5	37.3	0.8	2.7	107.0	2.7	30.0	1.1	100.0
18.3	4.5	0.9	1.9	143.0	2.7	30.0	0.6	114.0
26.7	26.2	1.1	1.9	152.0	2.7	30.0	0.5	81.0
49.8	27.2	0.8	19.5	113.0	2.7	30.0	0.9	101.0
25.4	20.4	1.0	2.0	123.0	2.7	30.0	0.8	107.0
25.8	24.3	1.0	2.0	138.0	2.6	30.0	0.5	87.0
21.6	14.4	0.8	1.9	147.0	2.6	30.0	1.6	112.0
23.2	23.5	0.9	1.6	150.0	2.6	30.0	1.2	143.0
21.7	18.9	0.8	1.8	133.0	2.6	30.0	0.6	70.0
18.1	11.5	0.6	2.0	142.0	2.6	30.0	0.8	89.0
27.7	17.5	1.0	2.5	138.0	2.6	30.0	1.4	176.0
21.0	21.0	0.9	1.5	123.0	2.6	30.0	1.4	141.0
27.2	11.2	1.1	2.5	116.0	2.6	30.0	1.6	122.0
25.8	29.3	1.1	1.7	127.0	2.6	30.0	2.2	46.0
20.2	18.8	0.9	1.4	158.0	2.5	30.0	2.0	101.0
39.5	19.8	2.6	3.1	149.0	2.5	30.0	1.1	129.0
21.4	27.7	0.8	1.6	149.0	2.5	30.0	1.7	148.0
22.5	19.0	0.8	1.8	123.0	2.5	30.0	1.7	171.0
23.3	24.7	1.0	1.6	126.0	2.5	30.0	1.1	91.0
31.2	15.9	1.1	3.3	122.0	2.5	17.0	1.7	96.0

49.6	32.4	0.7	19.4	130.0	2.4	14.0	1.3	134.0
20.5	17.4	0.7	1.7	134.0	2.4	30.0	0.5	71.0
17.4	10.5	1.1	1.1	126.0	2.4	30.0	0.8	104.0
20.8	17.4	0.7	1.9	112.0	2.4	30.0	1.1	169.0
19.8	33.8	0.8	1.2	133.0	2.4	30.0	1.1	136.0
18.7	9.8	1.2	1.2	54.0	2.4	30.0	1.3	116.0
18.9	26.7	0.8	1.2	110.0	2.4	30.0	1.2	168.0
25.5	19.1	0.9	2.3	81.0	2.4	10.0	0.7	50.0
23.2	21.2	0.9	1.8	111.0	2.3	30.0	1.9	99.0
24.8	19.9	0.8	2.4	73.0	2.3	30.0	1.1	55.0
28.3	20.6	0.8	3.2	108.0	2.3	30.0	0.4	56.0
25.1	27.9	0.9	1.8	120.0	2.3	30.0	1.4	150.0
15.2	0.9	1.6	1.6	109.0	2.3	9.0	1.2	54.0
21.4	17.2	1.0	1.5	143.0	2.3	30.0	0.8	127.0
23.7	14.4	0.8	2.4	136.0	2.3	30.0	1.0	202.0
21.5	13.9	0.7	2.0	101.0	2.3	30.0	1.3	55.0
19.1	15.1	1.0	1.3	150.0	2.3	30.0	1.5	162.0
28.6	28.0	1.1	2.1	126.0	2.3	3.0	2.1	67.0
16.6	8.2	0.7	1.6	153.0	2.3	30.0	1.2	125.0
22.5	25.3	0.7	1.9	137.0	2.2	30.0	1.0	52.0
19.5	10.4	0.7	2.0	134.0	2.2	30.0	1.3	51.0
26.3	21.7	0.8	2.4	118.0	2.2	30.0	1.0	109.0
23.0	21.2	0.7	2.0	119.0	2.2	30.0	1.0	91.0
17.5	12.5	0.6	1.8	120.0	2.2	30.0	1.3	87.0
28.3	13.1	1.1	2.7	137.0	2.2	30.0	1.0	108.0
18.5	7.1	0.8	1.8	146.0	2.2	30.0	1.9	154.0
25.0	10.4	1.2	2.0	108.0	2.2	30.0	1.1	3.9
41.3	16.7	0.9	9.7	107.0	2.2	20.0	1.2	140.0
22.0	11.8	0.9	2.0	157.0	2.2	30.0	1.2	77.0
26.7	26.1	0.9	2.3	133.0	2.2	30.0	2.5	99.0
20.5	10.5	1.0	1.6	164.0	2.1	30.0	2.1	136.0
21.4	14.2	1.1	1.5	178.0	2.1	30.0	1.0	201.0
31.4	22.2	1.1	3.0	99.0	2.1	30.0	0.6	68.0
26.5	19.6	0.8	2.6	129.0	2.1	30.0	0.7	83.0
22.0	15.3	0.9	1.8	102.0	2.1	30.0	0.8	102.0
24.7	12.9	0.8	2.5	162.0	2.1	30.0	1.5	154.0
19.9	7.6	0.8	2.1	133.0	2.1	30.0	2.5	119.0
23.3	10.9	0.8	2.4	146.0	2.1	30.0	1.3	88.0
24.2	23.7	1.0	1.6	133.0	2.1	30.0	1.8	129.0
27.6	28.1	0.9	2.4	163.0	2.1	30.0	1.0	92.0
15.9	17.6	0.6	1.4	152.0	2.1	30.0	1.3	75.0
25.2	18.1	1.0	2.1	132.0	2.1	30.0	2.3	63.0
22.9	6.0	1.5	1.7	89.0	2.1	30.0	3.0	115.0
19.3	15.4	0.8	1.5	146.0	2.1	30.0	1.7	128.0
20.8	28.1	0.8	1.3	139.0	2.1	30.0	1.4	122.0
19.4	11.3	0.9	1.5	157.0	2.1	30.0	1.7	87.0

25.2	24.7	0.8	2.1	135.0	2.0	30.0	0.8	115.0
23.1	21.2	0.8	2.0	125.0	2.0	30.0	1.0	61.0
29.8	21.8	1.1	2.7	95.0	2.0	3.0	4.9	44.0
23.9	23.0	0.7	2.2	65.0	2.0	30.0	0.4	52.0
23.2	20.4	0.7	2.1	138.0	2.0	30.0	1.6	136.0
24.1	9.7	1.4	1.7	139.0	2.0	30.0	0.9	40.0
28.4	24.4	1.6	1.6	129.0	2.0	30.0	1.1	90.0
18.7	20.9	0.6	1.7	119.0	2.0	30.0	1.5	66.0
29.1	15.7	1.2	2.5	131.0	2.0	30.0	4.1	127.0
25.4	18.9	0.4	4.3	145.0	2.0	5.0	1.5	59.0
18.4	8.9	0.8	1.7	169.0	2.0	30.0	0.7	141.0
16.3	3.1	1.0	1.7	120.0	2.0	30.0	1.0	57.0
15.8	8.2	0.8	1.3	132.0	2.0	30.0	1.9	72.0
29.4	29.3	0.8	2.9	135.0	2.0	26.0	0.5	42.0
21.1	17.9	0.8	1.6	128.0	1.9	30.0	1.8	94.0
27.0	16.6	1.0	2.5	135.0	1.9	30.0	1.6	102.0
24.9	21.1	1.1	1.8	134.0	1.9	30.0	1.5	134.0
23.1	33.8	0.8	1.7	127.0	1.9	30.0	1.7	154.0
17.3	23.1	0.4	2.1	167.0	1.9	30.0	2.0	0.5
14.6	3.9	0.8	1.6	94.0	1.9	30.0	0.7	30.0
23.8	14.0	0.7	2.5	142.0	1.9	30.0	1.1	112.0
24.8	22.5	1.0	1.9	143.0	1.9	30.0	1.9	10.0
18.3	6.6	1.1	1.5	141.0	1.9	30.0	1.1	139.0
25.4	18.1	1.0	2.0	146.0	1.9	30.0	1.5	105.0
24.1	17.4	1.0	1.8	127.0	1.8	30.0	0.7	56.0
21.9	41.1	0.7	1.6	140.0	1.8	18.0	1.8	34.0
29.6	24.0	0.9	3.0	138.0	1.8	30.0	1.6	82.0
25.4	22.5	0.8	2.3	130.0	1.8	9.0	2.4	96.0
26.0	26.6	1.2	1.7	148.0	1.8	30.0	1.9	78.0
22.1	13.4	0.9	1.9	129.0	1.8	30.0	1.4	93.0
20.9	27.7	0.8	1.5	98.0	1.8	30.0	1.1	62.0
12.6	13.5	0.4	1.5	115.0	1.8	30.0	1.2	90.0
25.8	32.2	0.9	2.0	123.0	1.8	30.0	1.1	102.0
26.6	23.2	0.8	2.5	125.0	1.7	30.0	1.1	75.0
12.0	11.1	0.5	1.3	110.0	1.7	29.0	0.5	141.0
30.3	32.1	1.3	2.2	112.0	1.6	30.0	0.8	35.0
23.5	15.5	0.7	2.5	131.0	1.5	30.0	1.4	82.0
23.2	13.2	1.0	1.8	153.0	1.5	30.0	1.2	109.0
22.2	30.5	0.6	2.0	124.0	1.5	30.0	0.8	56.0
11.6	10.7	0.6	1.1	153.0	1.5	30.0	1.4	102.0
26.3	15.2	1.0	2.4	151.0	1.5	30.0	1.3	58.0
11.6	2.9	0.6	1.6	105.0	1.5	24.0	1.7	58.0
19.9	18.5	0.8	1.5	139.0	1.5	30.0	0.9	65.0
24.7	34.5	1.1	1.5	135.0	1.5	30.0	1.9	75.0
27.6	32.8	1.0	2.0	102.0	1.4	30.0	1.5	143.0
17.7	8.8	0.7	1.8	136.0	1.2	30.0	2.9	121.0

24.7	16.3	0.9	2.2	129.0	1.2	30.0	2.2	79.0
25.9	24.4	0.5	3.7	68.0	1.2	30.0	1.0	190.0
24.3	42.7	0.7	1.8	112.0	1.1	30.0	3.2	82.0
20.9	12.1	0.8	1.8	166.0	1.1	30.0	1.8	101.0
16.2	3.8	0.8	1.9	121.0	1.0	30.0	2.6	61.0
15.7	13.3	0.8	1.2	136.0	0.9	30.0	2.1	256.0
21.5	9.1	0.9	2.0	163.0	0.9	30.0	2.7	66.0
25.8	18.1	1.1	1.9	131.0	0.8	20.0	0.6	60.0
41.1	8.1	2.2	5.5	127.0	0.8	2.0	1.5	84.0

neutrophil	ammonia	ALT	AST	1, HBEAG -	logHBVDN/	AFP	NLR group (1<3,23-
14.2	178.0	151.0	459.0	1.0	3.2	66.9	3
23.8	74.0	54.0	88.0	1.0	4.7	24.5	3
22.1	99.0	85.0	86.0	2.0	3.0	6.4	3
14.8	82.0	109.0	194.0	2.0	3.0	12.4	3
12.9	51.0	270.0	474.0	1.0	5.2	7.3	3
20.1	60.0	13.0	38.0	1.0	7.5	48.5	3
27.0	14.0	93.0	230.0	1.0	3.0	3.1	3
8.3	67.0	143.0	244.0	1.0	3.0	790.5	3
7.4	28.0	85.0	111.0	1.0	3.0	2.5	3
15.0	104.0	52.0	191.0	2.0	3.0	16.5	3
13.3	71.0	1185.0	1083.0	1.0	3.0	6.3	3
10.5	20.0	118.0	237.0	2.0	3.0	16.0	3
2.9	70.0	192.0	353.0	2.0	6.5	265.1	1
6.5	59.0	144.0	132.0	1.0	7.2	5.0	3
14.5	50.0	1129.0	1054.0	1.0	3.0	119.7	3
13.0	54.0	189.0	80.0	1.0	5.1	13.1	3
16.9	131.0	13.0	44.0	1.0	3.0	16.4	3
3.9	100.0	1351.0	794.0	1.0	3.0	9.9	3
8.2	60.0	1837.0	2750.0	1.0	3.0	10.9	3
5.0	75.0	49.0	66.0	1.0	3.0	159.6	3
6.1	205.0	33.0	60.0	1.0	3.0	14.4	3
6.8	85.0	570.0	1869.0	1.0	3.0	0.9	3
10.7	65.0	79.0	117.0	1.0	5.5	50.1	3
6.5	74.0	497.0	349.0	2.0	3.0	16.4	3
12.8	86.0	613.0	182.0	2.0	4.8	12.0	3
14.9	56.0	201.0	129.0	1.0	3.0	89.1	3
21.9	46.0	724.0	957.0	1.0	3.5	181.0	3
13.4	185.0	1695.0	1210.0	1.0	3.0	2.8	3
15.1	32.0	91.0	131.0	1.0	3.0	132.6	3
9.5	127.0	335.0	234.0	2.0	4.4	23.8	3
8.8	136.0	229.0	653.0	1.0	4.6	31.7	3
15.1	124.0	275.0	248.0	1.0	3.0	22.5	3
17.8	131.0	413.0	338.0	2.0	3.0	4.1	3
11.0	49.0	148.0	701.0	1.0	3.0	265.1	3
13.6	129.0	232.0	212.0	2.0	3.0	9.8	3
9.0	113.0	476.0	266.0	1.0	3.0	8.2	3
23.9	38.0	1542.0	1481.0	1.0	3.0	269.1	3
8.2	102.0	388.0	351.0	1.0	5.8	54.0	3
13.8	179.0	3.0	3.0	1.0	3.0	135.1	3
13.8	95.0	179.0	93.0	1.0	7.7	135.1	3
14.4	34.0	166.0	128.0	1.0	3.0	8.4	3
12.9	52.0	864.0	178.0	1.0	3.0	130.9	3
26.2	102.0	44.0	87.0	1.0	3.0	55.7	3
9.3	206.0	110.0	120.0	1.0	3.0	106.7	3
4.2	40.0	39.0	100.0	2.0	4.7	54.0	3

10.5	72.0	25.0	132.0	1.0	3.0	3.7	3
8.1	96.0	215.0	157.0	2.0	3.0	15.0	3
9.7	99.0	33.0	46.0	1.0	3.0	20.0	3
6.8	50.0	135.0	119.0	2.0	3.5	130.2	3
7.5	136.0	621.0	444.0	1.0	3.0	22.9	3
9.4	160.0	77.0	183.0	1.0	3.0	20.5	3
4.6	56.0	308.0	197.0	1.0	3.0	12.0	1
10.9	127.0	102.0	79.0	1.0	5.4	22.3	3
12.5	101.0	535.0	249.0	1.0	5.7	435.0	3
8.1	57.0	673.0	2416.0	1.0	3.0	12.6	3
24.0	46.0	40.0	53.0	1.0	4.0	524.7	3
12.5	123.0	70.0	100.0	2.0	3.0	1.0	3
17.2	31.0	8.0	23.0	1.0	3.0	12.1	3
5.8	133.0	16.0	39.0	1.0	3.0	172.6	3
7.6	44.0	52.0	81.0	2.0	3.0	53.3	3
10.4	56.0	747.0	567.0	1.0	7.6	7.8	3
9.1	77.0	423.0	1344.0	1.0	7.7	2.1	3
5.6	105.0	572.0	459.0	1.0	3.0	71.6	3
20.1	129.0	98.0	112.0	1.0	3.0	34.0	3
4.6	327.0	93.0	104.0	1.0	3.0	245.1	3
3.5	59.0	90.0	96.0	1.0	8.2	228.9	3
10.0	74.0	184.0	461.0	1.0	3.0	3.8	3
11.9	73.0	166.0	161.0	2.0	3.2	33.2	3
5.3	30.0	33.0	23.0	2.0	3.0	39.5	3
6.7	61.0	245.0	243.0	1.0	7.2	36.1	3
8.8	252.0	522.0	448.0	1.0	3.0	2.3	3
4.8	71.0	45.0	75.0	2.0	3.0	11.0	3
8.2	144.0	554.0	800.0	1.0	3.0	130.3	3
9.4	68.0	681.0	315.0	2.0	3.0	26.1	3
22.0	25.0	35.0	205.0	2.0	3.0	1.8	3
5.8	27.0	46.0	247.0	1.0	6.0	34.6	3
5.5	212.0	1103.0	1174.0	2.0	3.0	29.7	3
9.2	60.0	418.0	429.0	1.0	3.0	5.0	3
7.4	86.3	1367.0	1083.0	1.0	7.7	9.7	3
8.2	77.0	134.0	118.0	1.0	3.0	46.1	3
6.0	121.0	85.0	123.0	1.0	3.0	2.0	3
7.2	42.0	305.0	414.0	1.0	5.5	16.7	3
13.2	145.0	2413.0	1845.0	1.0	3.0	16.0	3
5.4	110.0	645.0	790.0	2.0	6.9	280.4	3
10.1	115.0	232.0	331.0	1.0	3.0	278.6	3
5.2	40.0	58.0	91.0	1.0	3.2	25.5	3
6.4	66.0	172.0	280.0	2.0	3.0	103.6	3
10.1	59.0	522.0	495.0	1.0	3.0	13.9	3
11.2	60.0	96.0	222.0	1.0	3.0	67.3	3
8.1	83.0	647.0	1768.0	1.0	3.0	34.5	3
12.6	48.0	490.0	133.0	1.0	3.0	12.7	3

7.9	139.0	251.0	287.0	2.0	3.0	78.0	3
8.8	107.0	320.0	345.0	1.0	3.2	14.0	3
7.9	65.0	70.0	114.0	2.0	3.0	4.3	3
4.2	174.0	637.0	525.0	2.0	3.0	11.0	3
2.3	11.0	42.0	146.0	1.0	6.2	20.9	3
3.2	100.0	793.0	702.0	1.0	7.6	80.0	3
8.7	40.0	224.0	231.0	1.0	4.7	12.0	3
12.3	49.0	245.0	111.0	2.0	3.0	3.3	3
7.7	99.0	119.0	194.0	1.0	7.6	10.7	3
3.7	47.0	1263.0	1772.0	2.0	6.8	208.1	3
8.2	132.0	287.0	198.0	2.0	5.2	3.3	3
6.7	29.0	365.0	300.0	1.0	6.1	55.2	3
9.4	86.0	110.0	279.0	2.0	3.0	2.2	3
3.2	79.0	411.0	742.0	1.0	3.0	33.6	3
6.1	36.0	54.0	45.0	2.0	4.4	12.0	3
5.9	102.0	700.0	588.0	1.0	3.0	51.0	3
5.5	33.0	150.0	149.0	1.0	6.2	24.8	3
7.1	65.0	33.0	85.0	1.0	3.0	216.1	3
7.4	88.0	310.0	282.0	1.0	7.0	70.9	3
8.1	91.0	1079.0	409.0	1.0	3.0	956.9	3
4.9	100.0	189.0	259.0	2.0	3.6	35.5	3
6.2	81.0	169.0	222.0	2.0	3.0	14.0	3
11.2	61.0	79.0	134.0	2.0	5.3	16.0	3
8.5	105.0	70.0	89.0	1.0	3.6	13.3	3
7.8	46.6	120.0	169.0	2.0	5.6	60.1	3
5.5	118.0	123.0	763.0	1.0	3.0	2.2	3
19.1	62.0	21.0	55.0	2.0	3.0	65.3	3
7.0	27.0	1419.0	1612.0	1.0	6.6	16.0	3
5.2	139.0	438.0	160.0	1.0	3.0	3.3	3
6.2	25.0	301.0	485.0	1.0	5.3	12.0	3
12.4	304.0	205.0	1072.0	1.0	5.4	15.3	3
3.7	68.0	50.0	143.0	1.0	3.0	14.8	3
13.9	114.0	30.0	59.0	2.0	3.0	210.2	3
5.2	148.0	216.0	199.0	1.0	3.0	0.7	3
16.4	77.0	73.0	97.0	1.0	3.0	44.1	3
11.0	30.0	44.0	134.0	2.0	3.0	100.3	3
5.4	50.0	598.0	336.0	1.0	7.7	33.5	3
16.0	72.0	1644.0	1814.0	2.0	3.0	19.6	3
5.2	109.0	160.0	126.0	1.0	3.4	36.6	2
15.9	121.0	730.0	398.0	1.0	5.3	10.4	3
3.1	23.0	1101.0	1369.0	1.0	7.1	29.7	3
3.4	103.0	1503.0	1333.0	2.0	3.7	131.0	1
7.5	110.0	70.0	65.0	2.0	3.0	130.5	3
15.4	97.0	71.0	126.0	1.0	3.0	2.2	3
5.5	21.0	60.0	80.0	2.0	5.8	88.3	3
5.4	56.0	1586.0	1000.0	1.0	3.0	25.8	3

4.2	51.0	34.0	104.0	1.0	3.0	148.1	3
8.2	93.0	109.0	117.0	1.0	4.2	17.0	3
8.1	93.0	512.0	198.0	2.0	3.0	1.4	3
8.8	65.0	195.0	105.0	1.0	4.7	214.6	3
7.8	48.0	75.0	68.0	1.0	3.0	11.0	3
7.4	41.0	140.0	73.0	2.0	6.3	208.1	3
8.7	155.0	1354.0	937.0	1.0	6.8	34.0	3
11.8	66.0	99.0	147.0	1.0	6.8	26.8	3
2.4	71.0	75.0	111.0	1.0	3.0	110.6	3
15.1	24.0	162.0	271.0	1.0	6.3	53.2	3
10.2	156.0	189.0	231.0	2.0	3.0	14.6	3
6.1	112.0	142.0	132.0	1.0	8.1	36.2	3
8.8	46.0	109.0	93.0	2.0	4.4	15.0	3
12.0	79.0	161.0	100.0	1.0	3.0	14.6	3
9.6	69.0	170.0	156.0	1.0	6.9	2.1	3
7.2	41.0	175.0	151.0	1.0	7.8	216.1	3
5.9	210.0	80.0	86.0	2.0	3.0	528.9	3
5.2	36.0	55.0	69.0	2.0	5.5	11.0	3
15.6	53.0	30.0	79.0	1.0	3.0	2.5	3
8.5	174.0	1197.0	921.0	1.0	3.0	6.4	3
3.7	8.0	153.0	248.0	1.0	3.0	11.0	3
4.0	60.0	178.0	85.0	1.0	3.0	41.3	3
11.9	182.0	61.0	104.0	1.0	7.7	110.6	3
7.9	24.0	26.0	84.0	2.0	3.0	11.0	3
8.8	30.0	831.0	938.0	1.0	7.5	1.9	3
6.7	65.0	68.0	107.0	2.0	3.0	12.4	2
7.9	59.0	2246.0	1877.0	2.0	3.0	5.2	2
11.1	91.0	1281.0	709.0	2.0	7.7	39.9	2
26.5	40.0	254.0	249.0	1.0	3.0	8.8	3
3.0	137.0	256.0	207.0	1.0	7.5	9.0	2
7.2	87.0	144.0	142.0	1.0	3.0	25.1	2
7.4	190.0	56.0	44.0	1.0	7.7	14.0	2
11.1	#NULL!	57.0	125.0	1.0	3.0	3.3	3
5.9	100.0	279.0	414.0	1.0	3.2	3.6	2
6.5	47.0	1431.0	1456.0	2.0	3.5	20.3	2
8.3	61.0	174.0	350.0	2.0	3.0	11.0	3
2.5	49.0	22.0	53.0	1.0	3.0	6.7	3
9.3	252.0	155.0	156.0	1.0	3.0	1.9	3
15.8	125.0	32.0	38.0	1.0	3.0	22.8	3
2.2	96.0	281.0	184.0	1.0	3.0	7.4	2
7.2	49.0	696.0	1086.0	1.0	7.9	343.9	3
8.1	90.0	38.0	52.0	2.0	3.0	34.0	3
5.2	62.0	1220.0	1128.0	1.0	3.0	3.6	2
1.8	52.0	679.0	460.0	2.0	3.5	149.3	3
7.4	19.0	56.0	25.0	1.0	3.0	6.4	2
2.6	62.0	26.0	87.0	1.0	4.0	22.0	3

9.1	124.0	1567.0	1342.0	1.0	6.7	46.8	2
5.0	94.0	711.0	708.0	1.0	6.2	77.5	2
4.1	88.0	2122.0	1537.0	1.0	3.0	11.0	2
5.7	158.0	1846.0	1757.0	1.0	6.9	1.5	3
8.9	51.0	65.0	92.0	1.0	3.6	370.9	3
3.9	64.0	758.0	1057.0	1.0	3.0	2.2	3
6.4	#NULL!	985.0	1550.0	1.0	7.1	93.2	3
7.4	54.0	94.0	213.0	1.0	4.9	15.0	3
4.6	196.0	1723.0	525.0	2.0	3.0	58.4	2
7.7	39.0	845.0	1681.0	1.0	7.1	154.8	3
9.3	18.0	19.0	93.0	2.0	4.1	2.0	3
8.2	77.0	432.0	174.0	1.0	3.0	5.4	2
4.0	121.0	176.0	92.0	2.0	6.7	81.4	2
7.4	93.0	1242.0	332.0	1.0	3.0	15.0	2
7.3	121.0	99.0	93.0	2.0	3.0	121.9	2
5.3	70.0	41.0	45.0	2.0	7.4	187.0	2
6.5	126.0	475.0	482.0	1.0	7.6	11.0	3
3.6	50.9	331.0	267.0	2.0	3.7	294.3	3
4.9	131.0	47.0	174.0	2.0	3.0	18.6	2
9.3	140.0	145.0	170.0	1.0	3.0	48.6	2
5.3	36.0	1627.0	988.0	2.0	6.9	2.9	3
3.6	43.0	118.0	236.0	2.0	3.0	84.9	2
6.2	173.0	183.0	174.0	1.0	3.0	45.4	2
12.1	59.0	369.0	492.0	1.0	6.9	127.0	3
4.8	41.0	170.0	86.0	1.0	5.1	165.9	3
1.4	52.0	36.0	99.0	1.0	3.0	106.2	3
7.9	74.0	171.0	297.0	1.0	8.2	262.1	2
11.4	47.0	73.0	110.0	1.0	3.0	27.5	2
6.6	30.0	62.0	133.0	1.0	3.0	9.4	2
5.7	43.0	101.0	103.0	2.0	3.8	35.7	2
4.7	52.0	115.0	125.0	1.0	4.3	10.6	2
8.9	71.0	99.0	260.0	1.0	3.0	214.4	2
5.0	89.0	1204.0	464.0	2.0	5.2	62.6	2
10.0	109.0	1724.0	1107.0	1.0	3.0	16.0	2
11.4	116.0	932.0	589.0	1.0	3.0	15.3	2
4.8	60.0	35.0	31.0	1.0	3.7	12.0	2
3.9	142.0	104.0	95.0	1.0	3.0	327.6	2
5.8	50.0	24.0	62.0	1.0	3.0	139.6	2
7.1	#NULL!	27.0	57.0	1.0	3.0	29.2	2
4.5	60.0	275.0	250.0	1.0	5.0	7.1	2
4.4	#NULL!	39.0	62.0	1.0	3.0	12.1	2
6.1	69.0	206.0	288.0	1.0	6.6	0.8	2
3.4	#NULL!	#NULL!	#NULL!	1.0	3.2	58.9	2
6.4	59.0	56.0	54.0	1.0	6.9	2.4	2
4.8	81.0	1316.0	1442.0	1.0	6.3	9.3	2
5.8	27.0	118.0	175.0	2.0	3.0	25.2	2

3.2	35.0	80.0	152.0	1.0	3.7	643.2	2
5.5	70.0	902.0	833.0	2.0	6.0	30.0	2
3.7	85.0	46.0	76.0	1.0	4.3	196.7	2
4.5	31.0	212.0	347.0	1.0	4.5	422.4	2
4.4	41.0	86.0	119.0	1.0	7.6	422.4	2
10.8	51.0	107.0	683.0	1.0	3.0	16.0	2
5.6	39.0	80.0	161.0	1.0	5.8	397.3	2
2.5	50.0	21.0	77.0	1.0	3.7	11.4	2
3.1	37.0	1163.0	1094.0	2.0	3.0	31.2	2
3.3	88.0	212.0	256.0	1.0	3.0	64.3	2
3.4	72.0	131.0	81.0	1.0	3.9	558.5	2
4.8	111.0	1225.0	953.0	1.0	3.0	95.7	2
4.2	67.0	74.0	84.0	1.0	7.5	11.5	2
4.9	43.0	110.0	85.0	2.0	3.0	14.0	2
3.9	55.0	957.0	797.0	1.0	5.3	130.3	2
9.0	18.0	294.0	301.0	1.0	3.9	34.7	2
2.4	86.0	128.0	230.0	2.0	3.6	222.1	2
4.0	40.0	70.0	149.0	1.0	3.0	92.0	2
5.3	#NULL!	53.0	56.0	1.0	5.4	48.5	2
4.1	70.0	129.0	341.0	1.0	3.0	54.0	2
4.7	81.0	283.0	306.0	2.0	5.1	3.2	2
4.7	44.0	164.0	244.0	1.0	3.9	151.3	2
2.8	37.0	117.0	114.0	2.0	3.3	131.1	2
4.8	110.0	501.0	403.0	1.0	5.8	17.6	2
3.7	73.0	163.0	90.0	1.0	4.8	146.8	2
3.3	156.0	48.0	119.0	2.0	3.0	260.0	2
1.8	36.0	678.0	582.0	1.0	3.0	212.8	2
9.6	76.0	462.0	400.0	2.0	3.0	12.0	2
3.8	78.0	104.0	62.0	1.0	4.2	1.1	2
2.5	92.0	904.0	531.0	2.0	3.1	56.5	2
5.5	57.0	1809.0	1455.0	2.0	3.0	2.4	2
4.2	46.0	147.0	129.0	1.0	3.4	214.6	2
6.5	22.0	278.0	144.0	2.0	3.0	15.3	2
5.3	59.0	343.0	322.0	2.0	3.8	64.7	2
4.6	58.0	2234.0	1937.0	2.0	7.2	15.0	2
4.0	57.0	46.0	111.0	1.0	3.0	52.0	2
8.4	58.0	2202.0	1676.0	2.0	4.3	194.0	2
3.9	105.0	96.0	256.0	2.0	3.0	54.0	2
4.0	132.0	22.0	43.0	1.0	3.0	3.8	2
5.5	82.0	490.0	222.0	1.0	3.0	14.6	2
3.8	111.0	3197.0	3574.0	1.0	7.7	11.0	2
6.0	26.0	28.0	52.0	1.0	3.0	1015.0	2
7.5	85.0	2092.0	878.0	2.0	3.0	9.9	2
5.8	66.0	231.0	210.0	1.0	3.0	407.3	2
2.4	123.0	97.0	128.0	1.0	6.3	345.7	2
3.8	#NULL!	197.0	175.0	1.0	5.0	117.6	2

2.8	47.0	462.0	174.0	1.0	6.5	91.2	2
2.2	54.0	232.0	463.0	1.0	3.0	210.2	2
2.2	83.0	88.0	249.0	1.0	3.0	16.5	2
7.9	#NULL!	66.0	96.0	2.0	3.0	910.3	2
2.6	67.0	18.0	127.0	2.0	3.0	862.7	2
6.9	50.0	130.0	150.0	1.0	4.4	2.1	2
1.5	134.0	221.0	127.0	1.0	7.7	20.1	2
8.4	204.0	281.0	272.0	2.0	3.0	59.6	2
5.9	59.0	403.0	327.0	2.0	3.3	303.5	2
1.6	63.0	2530.0	5580.0	1.0	4.3	31.2	2
7.2	43.0	185.0	310.0	2.0	7.1	1.7	2
3.2	41.0	46.0	97.0	1.0	3.3	110.7	2
5.9	36.0	399.0	335.0	1.0	3.0	869.9	2
6.4	136.0	602.0	97.0	2.0	6.3	15.0	2
5.7	47.0	55.0	107.0	1.0	3.0	19.6	2
4.1	63.0	619.0	295.0	1.0	4.6	33.8	2
3.3	58.0	232.0	326.0	2.0	3.2	28.9	2
4.4	48.0	929.0	537.0	1.0	3.0	52.3	2
3.5	20.0	293.0	458.0	2.0	4.7	16.1	2
2.7	81.0	156.0	276.0	1.0	6.9	218.7	2
4.1	147.0	112.0	113.0	1.0	7.0	156.2	2
8.6	36.0	22.0	74.0	1.0	3.0	13.6	2
5.5	129.0	96.0	106.0	2.0	3.0	218.0	2
6.0	47.0	549.0	316.0	2.0	3.0	206.0	2
3.2	77.0	925.0	290.0	2.0	3.0	8.5	2
9.6	69.0	43.0	146.0	2.0	3.0	14.0	2
2.4	72.0	122.0	203.0	2.0	3.0	15.0	2
4.4	62.0	3536.0	2103.0	1.0	7.1	390.4	2
3.4	88.0	43.0	146.0	2.0	3.7	23.7	2
3.3	45.0	60.0	72.0	1.0	3.1	173.1	2
1.6	4.0	120.0	207.0	2.0	4.6	35.9	2
3.7	83.0	238.0	84.0	1.0	3.0	23.7	2
5.9	82.0	1521.0	1641.0	1.0	6.3	610.5	2
2.8	38.0	450.0	714.0	1.0	5.8	3.2	2
3.7	60.0	764.0	672.0	1.0	3.8	12.1	2
7.0	100.0	359.0	473.0	1.0	3.0	30.7	2
3.5	103.0	1112.0	1029.0	2.0	3.0	54.0	2
5.5	53.0	176.0	123.0	1.0	6.7	212.8	2
3.1	#NULL!	202.0	249.0	1.0	3.0	2.9	2
4.6	79.0	147.0	233.0	2.0	3.0	231.5	2
8.5	114.0	98.0	29.0	2.0	4.7	3.5	2
2.8	92.0	270.0	457.0	2.0	3.0	3.5	2
3.7	86.0	52.0	81.0	1.0	3.9	408.8	2
3.5	67.0	102.0	68.0	2.0	3.0	28.0	2
8.8	107.0	325.0	337.0	1.0	4.8	50.8	2
8.8	107.0	325.0	337.0	1.0	3.0	50.8	2

3.5	66.0	257.0	272.0	2.0	4.5	4.9	2
1.9	34.0	475.0	523.0	1.0	3.0	48.5	2
3.7	99.0	151.0	144.0	1.0	6.8	104.1	2
4.3	125.0	63.0	126.0	1.0	3.8	14.3	2
4.7	100.0	53.0	98.0	2.0	4.8	34.0	2
2.3	97.0	509.0	216.0	2.0	3.0	65.5	2
10.0	82.0	124.0	286.0	2.0	3.0	7.1	2
6.6	31.0	133.0	236.0	1.0	3.0	18.5	2
5.1	63.0	622.0	328.0	2.0	5.0	117.5	2
6.2	44.0	211.0	98.0	2.0	3.5	577.3	2
2.4	138.0	1283.0	710.0	1.0	3.0	21.2	2
3.4	#NULL!	130.0	106.0	1.0	3.2	4.4	2
5.3	104.0	322.0	551.0	1.0	7.5	56.9	2
6.8	113.0	1934.0	1068.0	1.0	4.1	10.7	2
4.6	56.0	521.0	359.0	1.0	3.5	21.3	2
4.7	36.0	29.0	113.0	2.0	6.1	3.4	2
6.5	69.0	306.0	74.0	1.0	7.0	34.5	2
3.4	38.0	346.0	272.0	2.0	7.0	7.0	2
1.9	93.0	60.0	92.0	1.0	3.0	31.7	2
1.4	25.0	127.0	256.0	1.0	3.0	97.2	2
2.4	82.0	1795.0	1543.0	2.0	4.7	35.0	2
4.5	115.0	50.0	133.0	1.0	3.5	59.1	2
2.8	79.0	1082.0	222.0	1.0	7.5	32.6	2
5.2	110.0	748.0	633.0	1.0	5.8	188.0	2
6.8	56.0	127.0	132.0	1.0	3.6	147.7	2
3.1	120.0	1614.0	1542.0	1.0	3.0	30.8	2
3.9	71.0	1630.0	1645.0	2.0	4.0	295.5	2
4.8	41.0	779.0	498.0	1.0	3.0	41.1	2
3.0	99.0	94.0	58.0	1.0	4.6	230.7	2
2.9	38.0	202.0	116.0	1.0	3.0	73.7	2
2.8	87.0	38.0	110.0	1.0	4.5	4.0	2
2.5	64.0	154.0	268.0	1.0	3.1	121.5	2
3.2	101.0	496.0	246.0	2.0	4.5	275.4	2
7.5	118.0	435.0	232.0	2.0	3.0	14.9	2
2.6	62.0	71.0	91.0	1.0	7.5	279.7	2
7.1	45.0	1229.0	371.0	1.0	3.0	40.8	2
2.7	125.0	389.0	205.0	1.0	5.1	4.5	2
3.1	105.0	393.0	115.0	2.0	3.6	5.0	2
3.1	47.0	192.0	208.0	1.0	7.4	138.0	2
3.8	171.0	127.0	254.0	2.0	4.2	2.7	2
3.1	64.0	144.0	146.0	1.0	4.8	11.9	2
2.5	157.0	116.0	144.0	1.0	3.4	79.3	2
4.5	112.0	2191.0	1390.0	2.0	7.0	148.3	2
4.6	51.0	358.0	220.0	1.0	3.0	208.1	2
4.8	42.0	222.0	60.0	1.0	3.0	56.1	2
2.7	26.0	1912.0	1570.0	2.0	3.0	29.3	2

2.6	73.0	69.0	176.0	1.0	4.1	18.7	2
7.0	85.0	135.0	1036.0	2.0	7.6	16.0	2
1.2	76.0	680.0	347.0	2.0	5.9	136.0	2
2.5	36.0	393.0	100.0	2.0	6.5	23.6	2
4.1	33.0	55.0	149.0	2.0	3.0	269.4	3
2.3	26.0	9.0	47.0	2.0	3.7	11.5	2
6.0	114.0	668.0	411.0	1.0	3.0	30.9	2
2.3	26.0	9.0	47.0	2.0	3.0	11.5	2
3.2	126.0	158.0	245.0	2.0	5.9	190.4	2
3.7	78.0	182.0	127.0	1.0	5.3	1.1	2
2.7	121.0	99.0	20.0	1.0	6.0	983.4	2
2.8	71.0	351.0	250.0	2.0	6.7	51.2	2
1.2	63.0	93.0	173.0	1.0	6.4	1.9	2
3.4	83.0	90.0	96.0	1.0	5.6	7.7	2
3.9	111.0	218.0	216.0	1.0	5.6	20.2	2
3.0	91.0	382.0	497.0	2.0	3.0	19.0	2
2.1	56.0	1649.0	1160.0	1.0	3.9	199.7	2
2.3	60.0	503.0	785.0	1.0	7.0	24.6	2
5.4	68.0	172.0	48.0	2.0	4.4	82.8	2
2.8	120.0	446.0	331.0	2.0	7.4	24.2	1
1.5	52.0	320.0	313.0	2.0	3.0	3.6	1
0.8	90.0	151.0	88.0	1.0	3.0	4.3	1
3.4	85.0	1244.0	1511.0	1.0	3.0	19.7	1
5.4	88.0	569.0	571.0	1.0	3.0	67.0	1
3.6	103.0	1850.0	2013.0	2.0	4.6	435.5	1
3.9	103.0	72.0	113.0	1.0	3.1	17.7	1
1.3	101.0	887.0	450.0	2.0	3.6	70.0	1
4.9	96.0	750.0	410.0	1.0	4.6	206.0	1
5.2	62.0	811.0	1091.0	1.0	7.4	558.6	1
2.0	41.0	47.0	72.0	2.0	3.0	67.3	1
3.3	47.0	62.0	135.0	1.0	3.0	27.4	1
2.8	38.0	129.0	67.0	1.0	6.0	326.5	1
4.3	181.0	146.0	689.0	1.0	6.2	72.1	1
3.7	36.0	64.0	102.0	1.0	6.1	142.4	1
2.5	22.0	733.0	492.0	1.0	4.1	79.5	1
2.5	98.0	721.0	1039.0	1.0	5.4	95.4	1
3.0	128.0	216.0	355.0	2.0	6.3	12.0	1
4.1	58.0	384.0	151.0	1.0	4.5	2.5	1
5.2	74.0	2642.0	2159.0	1.0	6.6	14.0	1
2.4	18.0	42.0	42.0	1.0	5.6	170.3	1
3.2	135.0	71.0	115.0	1.0	5.7	3.1	1
3.1	92.0	373.0	294.0	2.0	6.0	45.2	1
4.4	67.0	55.0	45.0	2.0	3.0	16.8	1
4.8	50.0	186.0	153.0	2.0	3.0	55.6	1
1.7	35.0	541.0	492.0	1.0	3.8	15.0	1
4.4	31.0	272.0	108.0	2.0	3.0	212.8	1

0.8	41.0	24.0	38.0	1.0	3.0	692.3	1
1.6	124.0	289.0	296.0	1.0	3.0	4.8	1
2.5	31.0	160.0	78.0	1.0	3.0	11.8	1
3.9	18.0	26.0	63.0	1.0	3.0	15.2	1
5.2	102.0	130.0	187.0	1.0	3.1	3.5	1
2.9	100.0	725.0	702.0	1.0	6.3	83.8	1
3.0	67.0	92.0	81.0	2.0	7.0	64.3	1
1.3	55.0	52.0	80.0	1.0	3.2	15.0	1
2.6	54.0	689.0	562.0	1.0	3.3	32.1	1
4.4	56.0	99.0	99.0	1.0	4.3	8.6	1
6.4	50.0	82.0	122.0	2.0	3.0	590.6	1
1.8	59.0	520.0	405.0	2.0	3.7	12.8	1
3.3	103.0	159.0	106.0	1.0	6.9	549.0	1
3.6	133.0	158.0	58.0	2.0	5.9	69.7	1
2.2	141.0	284.0	198.0	2.0	4.6	28.4	1
5.3	37.0	90.0	60.0	1.0	3.0	1021.0	1
3.2	29.0	996.0	353.0	1.0	3.0	575.1	1
4.7	60.0	236.0	490.0	2.0	3.0	16.0	1
4.5	40.0	2026.0	1761.0	1.0	4.9	34.0	1
2.0	72.0	96.0	105.0	2.0	7.7	377.8	1
2.7	132.0	1414.0	545.0	2.0	3.3	11.0	1
2.8	41.0	43.2	53.0	1.0	3.0	46.4	1
3.4	59.0	3180.0	2531.0	1.0	3.9	572.4	1
3.3	76.0	81.0	205.0	1.0	6.9	14.0	1
2.5	58.0	700.0	250.0	2.0	3.0	345.5	1
2.4	78.0	168.0	333.0	1.0	3.0	224.5	1
2.8	28.0	83.0	206.0	2.0	3.0	17.0	1
1.6	73.0	811.0	484.0	1.0	6.0	13.9	1
1.3	35.0	1277.0	1411.0	1.0	3.0	1.8	1
2.1	24.0	90.0	125.0	2.0	5.0	92.5	1
2.0	85.0	700.0	1587.0	2.0	5.3	136.2	1
1.3	98.0	109.0	193.0	1.0	3.5	2.2	1
3.8	54.0	1491.0	801.0	1.0	4.0	27.7	1
2.8	91.0	620.0	377.0	2.0	6.2	81.0	1
4.2	#NULL!	116.0	117.0	2.0	6.0	168.1	3
1.8	98.0	732.0	396.0	1.0	6.1	12.0	1
3.2	119.0	1546.0	974.0	1.0	5.1	8.8	1
3.7	90.0	806.0	486.0	1.0	5.9	7.8	1
3.8	60.0	454.0	442.0	1.0	3.0	156.8	1
5.1	46.0	247.0	183.0	1.0	3.0	1.1	1
5.0	79.0	446.0	399.0	2.0	4.1	196.2	1
2.4	41.0	2112.0	1630.0	1.0	3.0	15.0	1
3.9	68.0	375.0	95.0	2.0	3.2	2.7	1
4.2	29.0	1116.0	642.0	1.0	5.1	26.0	1
2.7	57.0	173.0	98.0	1.0	3.9	13.6	1
4.1	110.0	160.0	157.0	1.0	4.1	2.1	1

2.8	81.0	67.0	60.0	2.0	4.9	66.3	1
1.2	52.0	799.0	665.0	1.0	3.3	4.4	1
14.4	51.0	57.0	32.0	1.0	6.9	445.3	3
2.3	55.0	197.0	288.0	1.0	3.0	505.4	1
2.3	58.0	292.0	163.0	1.0	3.0	157.7	1
3.0	#NULL!	40.0	40.0	2.0	8.2	238.4	1
2.5	65.0	58.0	58.0	1.0	3.9	196.3	1
1.6	67.0	588.0	1357.0	2.0	3.3	66.7	1
4.1	120.0	194.0	134.0	2.0	4.8	210.2	1
2.2	37.0	83.0	122.0	1.0	4.3	3.4	1
0.8	62.0	65.0	122.0	2.0	3.4	73.6	1
3.0	47.0	363.0	232.0	1.0	3.0	29.4	1
2.7	70.0	40.0	45.0	2.0	3.0	187.2	1
1.7	51.0	588.0	229.0	1.0	3.8	106.5	1
2.1	132.0	665.0	429.0	1.0	3.5	4.9	1
2.7	59.0	1219.0	1343.0	1.0	3.0	504.7	1
3.1	57.0	101.0	142.0	1.0	3.0	14.0	1
4.8	120.0	643.0	371.0	2.0	3.0	188.5	1
2.5	52.0	171.0	250.0	1.0	5.4	78.0	1
2.0	24.0	375.0	547.0	2.0	4.9	14.0	1
2.0	54.0	623.0	403.0	1.0	5.7	29.9	1
2.1	97.0	96.0	97.0	1.0	6.5	12.0	1
1.9	66.0	224.0	193.0	2.0	3.2	14.6	1
2.1	76.0	167.0	122.0	2.0	3.0	29.0	1
2.1	65.0	1224.0	1035.0	2.0	3.6	180.6	1
3.5	48.0	185.0	138.0	2.0	4.3	61.1	1
2.3	32.0	31.0	50.0	1.0	3.0	67.4	1
2.3	28.0	293.0	200.0	1.0	3.0	11.0	1
2.4	36.0	1074.0	1269.0	1.0	7.7	16.0	1
5.0	48.0	226.0	297.0	2.0	4.4	81.2	1
4.2	110.0	1559.0	1505.0	2.0	6.1	156.8	1
1.9	50.0	2460.0	2654.0	2.0	5.8	92.6	1
1.1	72.0	199.0	104.0	2.0	3.6	23.4	1
1.3	51.0	79.0	102.0	1.0	3.9	500.6	1
1.5	17.0	160.0	237.0	2.0	5.1	12.0	1
2.9	76.0	725.0	198.0	1.0	4.1	14.0	1
5.3	76.0	253.0	199.0	1.0	3.0	64.4	1
2.5	140.0	392.0	213.0	1.0	3.0	1.0	1
3.3	94.0	1511.0	1021.0	2.0	4.6	11.1	1
1.8	81.0	2305.0	1996.0	1.0	4.5	41.4	1
2.5	23.0	38.0	58.0	2.0	3.0	244.2	1
4.7	67.0	260.0	276.0	2.0	4.9	8.9	1
6.1	22.0	31.0	95.0	1.0	3.3	11.0	1
3.2	44.0	485.0	596.0	1.0	6.2	11.0	1
2.5	54.0	120.0	124.0	1.0	4.9	214.4	1
3.3	90.0	1049.0	578.0	2.0	3.0	39.3	1

1.4	61.0	753.0	506.0	2.0	6.6	5.1	1
1.9	134.0	633.0	376.0	1.0	4.9	13.2	1
8.9	203.0	110.0	136.0	1.0	6.6	16.0	1
4.3	48.0	22.0	32.0	1.0	3.0	286.5	3
3.3	74.0	645.0	483.0	2.0	7.7	42.8	1
1.8	99.0	86.0	182.0	2.0	3.0	12.9	1
1.8	136.0	302.0	73.0	2.0	3.0	57.9	1
2.7	110.0	1136.0	1827.0	1.0	4.3	285.9	1
7.3	#NULL!	#NULL!	#NULL!	1.0	3.0	46.6	1
2.9	177.0	829.0	812.0	1.0	7.8	92.3	1
1.3	18.0	1375.0	1236.0	1.0	5.3	17.1	1
1.9	54.0	154.0	363.0	1.0	6.4	51.8	1
3.7	57.0	573.0	419.0	2.0	7.6	7.3	1
1.8	129.0	93.0	118.0	2.0	6.8	20.9	1
3.4	70.0	35.0	70.0	2.0	3.0	16.1	1
2.7	73.0	176.0	209.0	2.0	3.9	16.5	1
2.5	62.0	355.0	250.0	1.0	6.8	103.0	1
2.8	17.0	137.0	83.0	2.0	5.0	10.1	1
3.8	42.0	1129.0	688.0	1.0	3.1	21.8	1
1.3	26.0	35.0	60.0	1.0	3.0	111.6	1
1.9	64.0	965.0	968.0	1.0	3.7	74.5	1
3.5	89.0	74.0	87.0	2.0	3.0	110.6	1
2.1	77.0	1826.0	1656.0	1.0	7.5	13.0	1
2.6	#NULL!	#NULL!	#NULL!	1.0	7.0	573.3	1
1.2	35.0	58.0	100.0	2.0	4.9	171.1	1
3.0	42.0	171.0	147.0	2.0	3.0	19.1	1
2.7	51.0	421.0	348.0	1.0	4.5	125.2	1
4.3	55.0	346.0	220.0	1.0	4.9	1.0	1
3.0	11.0	506.0	1096.0	1.0	5.3	11.5	1
2.3	24.0	460.0	248.0	1.0	3.9	53.7	1
1.8	41.0	110.0	123.0	2.0	5.1	47.0	1
1.9	90.0	173.0	283.0	2.0	4.2	15.0	1
1.8	197.0	118.0	137.0	1.0	3.0	32.4	1
1.7	102.0	318.0	352.0	2.0	6.9	38.2	1
2.0	47.0	430.0	992.0	1.0	3.0	1.8	1
1.2	58.0	261.0	128.0	2.0	5.4	343.7	1
1.9	22.0	185.0	139.0	2.0	3.0	195.0	1
1.7	87.0	1990.0	1912.0	2.0	3.7	14.0	1
1.1	117.0	224.0	250.0	1.0	5.6	99.3	1
2.0	53.0	547.0	354.0	2.0	4.1	89.4	1
1.8	210.0	290.0	274.0	1.0	3.0	32.0	1
2.5	56.0	559.0	585.0	2.0	6.8	12.9	1
1.2	100.0	430.0	551.0	1.0	6.2	125.9	1
2.8	39.0	47.0	74.0	1.0	3.4	34.0	1
2.0	86.0	121.0	312.0	1.0	5.9	25.7	1
3.6	50.0	360.0	520.0	2.0	5.7	1.1	1

2.7	47.0	42.0	51.0	1.0	3.0	28.3	1
1.1	87.0	22.0	42.0	1.0	7.0	301.8	1
3.1	61.0	160.0	85.0	1.0	3.3	53.2	1
1.8	77.0	674.0	552.0	2.0	6.5	157.1	1
2.5	52.0	109.0	210.0	1.0	4.4	472.5	1
1.8	76.0	561.0	548.0	2.0	6.8	4.6	1
2.3	86.0	289.0	184.0	2.0	5.6	16.0	1
4.6	50.0	52.0	100.0	2.0	3.0	214.4	3
11.0	147.0	4664.0	5720.0	1.0	3.0	17.6	3

Antiviral	Antiviral drug 1ADV 2 ETV 3 LAM 4 LDT 5 ADV+ETV/LAM+ADV 6 IFN
1	2
2	0
1	2
1	3
1	2
1	2
1	3
1	3
1	2
1	2
1	2
2	0
1	2
1	5
1	2
1	1
1	3
1	3
1	5
1	2
1	1
1	3
1	2
1	2
1	5
1	1
1	2
1	5
1	2
1	2
1	3
2	0
1	2
1	2
1	3
1	3
1	1
1	2
2	0
1	2
1	6
1	3
1	3
1	2
1	1

1	4
2	0
1	2
1	3
1	2
1	6
1	1
1	5
1	3
1	2
1	1
1	3
1	2
1	3
1	3
1	3
1	2
1	1
2	0
1	2
1	3
1	2
1	3
1	5
1	1
1	2
1	3
1	3
1	1
1	2
1	2
1	2
2	0
1	3
1	1
1	1
1	2
1	2
1	2
1	3
1	2
1	2
2	0
1	1
1	2
1	2

1	5
1	2
1	3
1	2
1	3
1	3
1	2
1	3
1	2
1	1
1	3
1	3
1	5
1	2
1	2
1	3
1	3
1	1
1	2
1	1
1	1
1	3
1	1
1	2
1	3
1	3
1	3
1	3
1	2
1	3
1	4
1	5
1	3
1	2
1	2
1	4
1	1
1	2
1	3
1	5
1	6
1	2
2	0
1	1
1	1
1	3

1	1
1	3
1	2
1	3
1	5
1	3
1	3
1	1
1	2
1	3
1	2
1	2
1	2
1	2
2	0
1	2
1	2
1	1
1	5
1	1
1	3
1	3
1	3
1	3
1	2
1	3
1	5
1	5
1	2
1	2
1	2
1	2
2	0
1	3
1	2
1	2
1	3
1	3
1	1
1	3
1	1
1	2
1	2
1	1
1	2
1	2

1	2
1	2
1	2
1	3
1	2
1	2
1	1
1	5
1	3
1	3
1	3
1	4
1	1
2	0
1	2
1	1
1	6
1	1
1	2
1	1
1	2
1	3
1	2
1	3
1	2
1	2
1	3
1	2
1	5
1	3
1	2
2	0
1	2
1	3
2	0
1	2
1	2
1	2
1	2
1	3
1	2
1	2
1	3
1	2
1	1
1	2

1	5
1	3
1	2
1	2
1	1
1	2
1	2
1	1
1	3
1	3
1	5
1	2
1	5
1	3
1	3
1	2
1	3
1	3
1	2
1	3
1	1
1	1
1	3
1	2
1	3
1	3
1	3
1	1
2	0
1	3
1	6
1	2
1	3
1	3
1	1
1	3
1	1
1	5
1	5
1	1
1	3
1	3
1	3
1	2
1	1
1	4

1	3
1	2
1	2
1	2
1	1
1	3
1	3
1	3
1	3
1	2
1	2
1	1
1	2
1	2
1	2
1	5
1	3
1	2
1	2
1	3
1	1
1	1
1	2
1	5
1	5
1	1
1	1
1	2
1	1
1	5
1	3
1	2
1	3
1	3
1	3
0	2
1	1
1	2
2	0
2	0
1	1
1	3
1	2
1	5
2	0
2	0

1	2
1	3
1	2
1	1
1	5
1	5
1	3
1	2
1	5
1	3
1	1
1	3
1	3
1	2
1	3
1	1
1	2
1	2
1	3
1	1
1	2
1	2
1	1
1	1
1	3
1	5
1	2
2	0
1	3
2	0
1	2
1	2
1	1
1	2
1	2
1	2
1	3
1	1
1	2
1	3
1	2
1	3
2	0
1	2
1	5
1	5

1	2
2	0
1	3
1	2
1	1
1	2
1	3
1	2
1	1
1	5
1	3
1	1
1	2
2	0
1	2
1	2
1	6
1	3
1	2
1	3
1	3
1	3
1	1
1	1
1	3
1	3
1	2
1	2
1	1
1	2
1	1
1	1
1	1
1	3
1	1
1	3
1	2
1	1
1	1
2	0
1	3
1	2
1	3
1	2
1	2
1	2

1	2
1	2
1	3
1	1
1	2
1	2
1	2
1	2
1	1
1	4
1	1
1	2
1	3
1	2
1	3
1	2
1	3
1	2
1	3
1	3
2	0
1	2
1	1
1	2
1	5
1	2
1	3
1	3
1	2
1	3
1	2
1	1
1	5
1	2
1	2
1	2
1	1
1	2
1	3
1	3
1	1
1	3
1	2
1	2
1	1
1	2

1	2
1	1
1	1
1	2
1	2
1	1
1	3
1	3
1	1
1	1
1	2
1	2
2	0
1	3
1	2
1	3
1	3
1	6
1	2
1	2
1	5
1	2
1	4
1	2
2	0
1	1
1	2
2	0
1	2
2	0
1	6
1	2
1	2
1	1
1	2
1	2
1	2
2	0
1	5
1	1
1	1
1	3
1	2
1	1
1	2
1	2

1	2
1	2
1	1
1	3
1	0
1	3
1	3
1	3
1	3
2	0
1	2
1	2
1	2
1	5
1	1
1	2
1	2
1	2
1	1
1	2
1	1
1	2
1	3
1	2
1	3
1	5
1	2
1	2
2	0
1	1
1	1
1	1
1	2
1	4
1	2
1	2
1	3
1	1
1	2
1	1
1	1
1	2
1	2
1	3
1	2
1	2

1	3
1	2
1	1
1	2
1	2
1	1
1	2
1	1
1	2
